# Decreased AMPK/SIRT1/PDK4 induced by androgen excess inhibits human endometrial stromal cell decidualization in PCOS

**DOI:** 10.1007/s00018-024-05362-5

**Published:** 2024-07-30

**Authors:** Ling Hong, Shan Xiao, Lianghui Diao, Ruochun Lian, Cong Chen, Yong Zeng, Su Liu

**Affiliations:** 1 Shenzhen Key Laboratory of Reproductive Immunology for Peri-Implantation, Shenzhen Zhongshan Institute for Reproductive Medicine and Genetics, Shenzhen Zhongshan Obstetrics and Gynecology Hospital (Formerly Shenzhen Zhongshan Urology Hospital), Shenzhen, China; 2 Guangdong Engineering Technology Research Center of Reproductive Immunology for Peri-Implantation, Guangdong, China

**Keywords:** Polycystic ovary syndrome, Hyperandrogenism, Decidualization, Pyruvate dehydrogenase kinase 4

## Abstract

Polycystic ovary syndrome (PCOS) is a complex common endocrine disorder affecting women of reproductive age. Ovulatory dysfunction is recognized as a primary infertile factor, however, even when ovulation is medically induced and restored, PCOS patients continue to experience reduced cumulative pregnancy rates and a higher spontaneous miscarriage rate. Hyperandrogenism, a hallmark feature of PCOS, affects ovarian folliculogenesis, endometrial receptivity, and the establishment and maintenance of pregnancy. Decidualization denotes the transformation that the stromal compart of the endometrium must undergo to accommodate pregnancy, driven by the rising progesterone levels and local cAMP production. However, studies on the impact of hyperandrogenism on decidualization are limited. In this study, we observed that primary endometrial stromal cells from women with PCOS exhibit abnormal responses to progesterone during in vitro decidualization. A high concentration of testosterone inhibits human endometrial stromal cells (HESCs) decidualization. RNA-Seq analysis demonstrated that pyruvate dehydrogenase kinase 4 (PDK4) expression was significantly lower in the endometrium of PCOS patients with hyperandrogenism compared to those without hyperandrogenism. We also characterized that the expression of PDK4 is elevated in the endometrium stroma at the mid-secretory phase. Artificial decidualization could enhance PDK4 expression, while downregulation of PDK4 leads to abnormal decidualization both in vivo and in vitro. Mechanistically, testosterone excess inhibits IGFBP1 and PRL expression, followed by phosphorylating of AMPK that stimulates PDK4 expression. Based on co-immunoprecipitation analysis, we observed an interaction between SIRT1 and PDK4, promoting glycolysis to facilitate decidualization. Restrain of AR activation resumes the AMPK/SIRT1/PDK4 pathway suppressed by testosterone excess, indicating that testosterone primarily acts on decidualization through AR stimulation. Androgen excess in the endometrium inhibits decidualization by disrupting the AMPK/SIRT1/PDK4 signaling pathway. These data demonstrate the critical roles of endometrial PDK4 in regulating decidualization and provide valuable information for understanding the underlying mechanism during decidualization.

**Supplementary Information:**

The online version contains supplementary material available at 10.1007/s00018-024-05362-5.

## Introduction

Polycystic ovary syndrome (PCOS) is a metabolic and endocrine disorder that majorly affects around 4–21% of women at reproductive age [1], which is characterized by oligo- or anovulation, hyperandrogenism (clinical or biochemical), and polycystic ovarian morphology (PCOM) on ultrasound [2]. Ovulatory dysfunction is considered to be a major cause of infertility related to PCOS. However, even ovulation is pharmacologically restored, anovulatory PCOS patients still have reduced cumulative pregnancy rates and exhibit a higher rate of spontaneous miscarriage [3]. Moreover, it was observed that the donated oocytes from PCOS women did not affect the implantation rate in the respective recipients [4]. These findings indicate that there are other determinants contributing to PCOS infertility, such as endometrial factors. An increasing number of studies have revealed several endometrial characteristics/markers related to the PCOS phenotype possibly explaining some of the unfavorable endometrium-related clinical manifestations. Adverse effects of hyperandrogenism, insulin resistance, or chronic inflammation can be possible mechanistic approaches for dysregulation of molecular or biochemical cascade requisite for endometrial receptivity in PCOS patients [5]. These aberrations can impede endometrial growth, decidualization, and placentation leading to pregnancy complications. However, the precise pathogenesis of endometrial dysfunction leading to implantation failure or pregnancy loss in PCOS patients is still elusive.

Hyperandrogenism is a common clinical manifestation in PCOS, which may lead to implantation failure, increased risk of miscarriage, adverse pregnancy outcomes, and endometrial disorders [6]. Androgens could promote uterine growth through androgen receptor (AR) [7]. Experimental findings derived from AR-knockout female mice showed the pivotal role of AR in uterine morphology, as evidenced by the observation of diminished uterine walls and reduced uterine area [8]. The AR expression is confined to the endometrial stroma and fluctuates during the menstrual cycle with a gradual decrease from the proliferative to the mid-secretory phase [9]. Furthermore, enzymes involved in the biosynthesis and conversion of androgens display elevated activity during the secretory phase [10], suggesting a crucial role of androgens in the process of decidualization. Recent investigations have demonstrated that androgens, including testosterone and dihydrotestosterone (DHT), could enhance the expression of prolactin (PRL) and insulin-like growth factor binding protein 1 (IGFBP1) and promote the morphological and ultrastructural changes of human endometrial stromal cells (HESCs) [11]. Nevertheless, a comprehensive understanding of the precise mechanisms underpinning the involvement of hyperandrogenic stimulation in decidualization of PCOS patients remains an area warranting further investigation and clarification.

Decidualization is the differentiation of elongated, fibroblast-like mesenchymal cells into rounded, epithelioid-like cells [12], and the morphological differentiation and hypermetabolic state of HESCs prepare them for the crucial event of embryo implantation [13–15]. Aberrant decidualization may lead to placental distortion and adverse pregnancy outcomes. It is worth noting that androgen excess inhibits the proliferation and differentiation of HESCs through AR [16], which is increased in the endometrium of PCOS [6]. This heightened the presence of AR amplifies the influence of androgen on the endometrium, leading to a poor endometrial receptivity, infertility, and spontaneous abortion [17]. It has been reported that hyperandrogenism decreases the expression of endothelial growth factors, thereby inhibiting the growth and development of the endometrium [17].

Glucose metabolism is important for the preparation of the endometrial stroma during early pregnancy [18]. During decidualization, the expression of various glycolysis-related enzymes and lactic acid was significantly increased, and glucose transporter 1 (GLUT1) and phosphorylated protein kinase B (AKT) were highly expressed in the decidual region due to the decreased oxygen consumption and increased glucose uptake [5, 12]. In human endometrial stromal cells, the knockdown of pyruvate kinase M2 (PKM2) and other glycolysis-related metabolic enzymes significantly inhibits estrogen-induced cell proliferation and lead to decidualization damage [6, 9, 19]. Among the 740 differentially expressed genes (DEGs) between PCOS and controls in our previous study [20], pyruvate dehydrogenase kinase 4 (PDK4) was the most salient gene related to pyruvate metabolism and glycolysis, which obstructs pyruvate dehydrogenase-mediated fueling of glucose-derived pyruvate oxidation into the tricarboxylic acid (TCA) cycle [21]. However, little is known regarding the role of PDK4 during the process of decidualization. We speculated that PDK4 may be involved in the abnormal decidualization during early pregnancy in PCOS.

In this study, we hypothesized that androgen excess is involved in the pathogenesis of infertility in PCOS. Our results showed that aberrantly increased androgen exerts a detrimental influence on decidualization, mainly by regulating the expression of PDK4. We demonstrated that androgen excess decreases the secretion of PRL and IGFBP1 and inhibits cytoskeletal reorganization during decidualization. Furthermore, our investigation elucidated that androgen excess exerts an inhibitory effect on PDK4 through a well-defined pathway, involving AR and AMP-activated protein kinase (AMPK). Notably, Silent information regulator factor 2-related enzyme 1 (SIRT1) emerges as an upstream regulator of PDK4 in this regulatory cascade, proposing a possible mechanism to explain how decidualization is affected by androgen excess and glucose utilization under abnormal endometrial conditions.

## Materials and methods

### Study population

## Ethical approval was obtained from the Institutional Ethics Committee of Shenzhen Zhongshan Obstetrics and Gynecology Hospital (formerly Shenzhen Zhongshan Urology Hospital), and the ethical approval number was SZZSECHU-F-2020023. All participants were aware of the research objective and signed informed consent forms.

In this study, the PCOS cases were extracted from the screening database according to the Rotterdam criteria, which was used for the diagnosis of PCOS based on the association of at least two of the three following criteria: oligo/anovulation, clinical or biochemical hyperandrogenism, and polycystic ovaries at ultrasonography. The controls were recruited voluntarily from the patients examined during the study period. The controls attended the clinic for the in vitro fertilization/intracytoplasmic sperm injection (IVF/ICSI) and the sole cause of marital infertility was male azoospermia based on clinical evaluation. All controls had regular menstrual cycles and normal androgen levels, none had polycystic ovaries on ultrasound. The subjects with endometriosis, endometritis, autoimmune- or thyroid-related disease, abnormal karyotypes, positive infectious disease tests, uterine malformation, and ultrasonographic evidence of hydrosalpinx were excluded from the control group. It is worth noting that the assessment of fasting glucose and fasting insulin levels is typically conducted among patients diagnosed with PCOS, individuals with obesity, or those who have metabolic abnormalities in our center. Consequently, the absence of these measurements in the control group precluded the comparative analysis. No subjects had received hormonal treatment or insulin-lowering agents in the previous quarter.

The clinical characteristics of PCOS patients and controls whose endometrium samples were used in this study are listed in Supplemental Table 1. In this study, we analyzed the transcriptional profiles between PCOS patients with hyperandrogenism and those without hyperandrogenism. The patients’ clinical details between PCOS with hyperandrogenism (H_PCOS) and PCOS without hyperandrogenism (N_PCOS) are displayed in Table 1.

**Table 1:** Characteristics of PCOS with and without hyperandrogenism

Characteristics	N_PCOS (*n* = 5)	H_PCOS (*n* = 5)	PCOS (*n* = 10)	*p* value^a^
Age (year)	31.40(30.0–36.0)	28.60(25.0–34.0)	30.00(25.0–36.0)	0.447
BMI (kg/m^2^)	24.7(19.9–29.6)	22.9(19.5–27.4)	23.8(19.5–29.6)	0.198
FSH (IU/L)	6.65(4.26–10.83)	6.64(3.63–8.93)	6.65(3.63–10.83)	0.996
LH (IU/L)	6.60(3.88–9.71)	10.89(4.98–18.81)	8.75(3.88–18.81)	0.227
LH/FSH	1.02(0.80–1.60)	2.01(0.56–4.52)	1.52(0.56–4.52)	0.246
Total T (ng/mL)	0.28(0.14–0.37)	0.69(0.50–0.86)	0.49(0.14–0.86)	0.001
TSH (μIU/mL)	2.50(1.89–3.19)	2.49(1.25–3.88)	2.50(1.25–3.88)	0.422
Fasting glucose (mmol/L)	5.44(4.95–6.20)	5.40(5.01–6.09)	5.42(4.95–6.20)	0.906
Fasting insulin (μU/mL)	10.0(5.0–14.0)	14.4(10.4–21.9)	12.2(5.0–21.9)	0.159
AMH (ng/mL)	3.79(3.35–4.02)	3.97(2.63–5.92)	3.89(2.63–5.92)	0.980

*BMI* body mass index, *FSH* follicle stimulating hormone, *LH* luteinizing hormone, *T* testosterone, *TSH* thyroid stimulating hormone, *AMH* anti-mullerian Hormone

^a^N-PCOS vs. H-PCOS

### Endometrial sample collection

Hormone replacement therapy cycles were used for enrolled anovulatory PCOS patients, during which estrogen and progesterone were administered consecutively to mimic the endocrine conditions of the endometrium in the natural menstrual cycle. The secretory endometrium was obtained after daily progesterone injections for 6–8 days in anovulatory women with PCOS, based on published methods that mimic mid-secretory endometrium [22]. Endometrial tissue samples from controls were collected during the mid-secretory phase of the menstrual cycle between day 7 and day 9 after the luteinizing hormone (LH) surge. Endometrial biopsies were obtained by soft curettage using an endometrial curette and processed within 2 h after collection. The endometrial samples were divided into two pieces: one was subjected to routine paraffin embedding, and the other was snap-frozen in liquid nitrogen and stored in a deep freezer until RNA extraction. Paraffin-embedded endometrial tissues were sectioned at 4 μm thickness. For each sample, one section was randomly chosen for H&E staining to confirm the histologic phase.

### RNA sequencing

The mRNA expression profiles of 12 controls and 12 individuals with PCOS were analyzed by applying the Affymetrix GeneChip PrimeViewTM Human Gene Expression Array (Thermo Fisher Scientific, Meridian, USA). Data from the Gene Expression Omnibus (GEO) database (series GSE103465) were analyzed. The *P*-value between the two groups was automatically generated with the limma algorithm in the R package, which uses a linear-regression model and Bayes testing for DEGs screening. Based on this previous study, we further analyzed the transcriptional profile of endometrium in H_PCOS and N_PCOS.

### Isolation of human primary endometrial stromal cells and decidualization in vitro

Human endometrial stromal cells were isolated from endometrial tissues collected by endometrial biopsies. Briefly, endometrial tissues were rinsed in DMEM/F-12 medium and minced. Minced tissues were incubated with collagenase type 4 (1 mg/mL) (Worthington, America, LS004189) and deoxyribonuclease type 1 (12500 μg/L) (Roche, Germany, 25,761,700) in 5 mL DMEM/F-12 medium (Gibco, USA, 11,330,032) for 1 h at 37 °C with manual agitation at a 20 min interval. After digestion, tissues were filtered through 100 μm and 40 μm cell strainers to eliminate tissue fragmentation and glandular clumps and flushed with DMEM/F-12 containing 10% certified fetal bovine serum (FBS) (Gibco, USA, 10,099,158). Cells were collected by centrifugation (1000 rpm, 5 min) and re-suspended in a DMEM/F-12 medium containing 10% FBS for culture. The medium was changed 6-18 h post-seeding to remove unattached epithelial cells, red blood cells, and immune cells. Cells were cultured with 0.5 mM 8-Bromo cyclic adenosine monophosphate (8-Br-cAMP) (Sigma, USA, B5386) and 1 μM medroxyprogesterone acetate (MPA) (Sigma, USA, M1692) to induce decidualization in vitro. Then, differentiation was evaluated by assessing the morphology and the expression of specific markers after varying durations of treatment with 8-Br-cAMP and MPA, ranging from 1 to 7 days.

### Cell culture and siRNA transfection

The human endometrial stromal cells (HESCs) were purchased from ATCC (CRL-4003TM). HESCs were cultured in a DMEM/F-12 medium supplemented with 10% FBS (Gibco, USA, 10,099,158), 1% penicillin/streptomycin (Gibco, USA, 15,140,122), 1% Insulin-Transferrin-Selenium (ITS-G) + Premix (ThermoFisher, USA, 41,400,045), and 500 ng/mL puromycin (Invitrogen, France, A1113803). It should be emphasized that charcoal/dextran treated FBS, which is effective in lowering steroid levels according to the ATCC’s recommendation, was not used in the endometrial stromal cell lines. Cells were placed in a 37 °C, 5% CO_2_ concentration incubator. For particular experiments, HESCs were pretreated with 0.5 mM 8-Br-cAMP and 1 μM MPA for 3 days, followed by treatment with testosterone (Sigma-Aldrich, USA, 58–22-0) or various signaling molecules’ inhibitor/activator for an additional 2 days, including Sodium dichloroacetate (DCA) (Selleck, USA, S8615), A-769662 (Abcam, UK, ab120335), Dorsomorphin (Compound C) (Abcam, UK, ab120843), SRT1720 (Abcam, UK, ab273598), EX527 (Abcam, UK, ab141506), and ARV-110 (Selleck, USA, S6965). It is important to note that during two-day treatment with testosterone or other molecules, 8-Br-cAMP and MPA were not included and the cells were treated exclusively with testosterone or other molecules. HESCs were transfected with each siRNA (100 pM) by Lipofectamine 3000 (Invitrogen, Carlsbad, CA) as per the manufacturer’s instruction. 4 μL of si-PDK4 (Sangon, China)/si-PDHE1A (Sangon, China) or scrambled control (Sangon, China) and 2 μL of Lipofectamine 3000 were separately diluted with 50 μL DMEM/F-12 medium for 5 min and mixed immediately at room temperature away from light. The compounds were added to orifice plates at equal levels. Penicillin/streptomycin-free DMEM/F-12 medium was added to make the total volume 500 μL. Cells were collected 2 days after treatment for further quantitative real-time PCR (qRT-PCR), western blot analysis, and immunofluorescence staining. The sequences of si-PDK4, si-PDHE1α and scrambled control were designed and synthesized by Sangon Biotech (Shanghai, China). The sequences of si-PDK4 are 5′-GUUCGAAAUAGACACCAUAAUTT-3′ (sense) and 5′-AUUAUGGUGUCUAUUUCGAACTT-3′ (antisense), and the sequences of si-PDHE1α used for knock-downing the expression of PDHE1α are 5′-CCAAGCCACAUUGGAAGCAUUTT-3′ (sense) and 5′- AAUGCUUCCAAUGUGGCUUGGTT-3′ (antisense). The scrambled sequences for control are 5′-UUCUCCGAACGUGUCACGUTT-3′ (sense) and 5′-ACGUGACACGUUCGGAGAATT-3′ (antisense) for HESCs.

### RNA extraction and quantitative real-time PCR

Total RNA was extracted from 500 ng of endometrium or treated HESCs using TRIzol reagent (ThermoFisher, USA, 15596026CN) and was used for cDNA synthesis, which was performed using the M-MLV reverse transcriptase kit (Toyobo, Japan) according to the manufacturer’s instructions. Synthesized cDNA was utilized for PCR with primers at optimized cycles (Supplemental Table 2). qRT-PCR analysis was performed using the ABI QuantStudio^TM^5 Real-Time PCR System (Applied Biosystems, CA, USA) with the Luna® Universal qPCR Master Mix Kit (New England Biolabs, Beijing, China). For comparison of transcript levels between samples, a standard curve of cycle thresholds for several serial dilutions of a cDNA sample was established and then used to calculate the relative abundance of each gene. Values were then normalized to the relative amounts of β-actin. All experiments were performed at least in triplicate for each gene.

### Western blot

Whole lysates from endometrium or treated HESCs were extracted with RIPA buffer (Beyotime, China, P0013B) containing a protease inhibitor cocktail (Beyotime, China, P1005). Protein concentrations were determined using the BCA Protein Assay Kit (Beyotime, China, P0012). A total protein assay was conducted for data normalization and comparison on the Protein Simple Wes™ Western Blot (WES) system (ProteinSimple, USA) according to the manufacturer’s instructions with a 12–230 kDa separation module (ProteinSimple, USA, SM-W004) and total protein detection module (ProteinSimple, USA, DM-TP01). The proteins were resolved to perform a western blot analysis on the Protein Simple Wes™ Western Blot machine (ProteinSimple, USA) with antibodies against the proteins of interest, including PDK4 (1:200, Abgent, AP7041B), AR (1:100, Invitrogen, AN1-15), phosphor-AMPK (p-AMPK) (1:200, cell signaling technology, 2535S), SIRT1 (1:100, cell signaling technology, 8469S) using a standard procedure, and the expression of each protein was normalized to the expression of Vinculin in the corresponding sample.

### Immunohistochemistry and multiplex immunostaining

Endometrial tissues were collected during the mid-secretory phase using an endometrial curette. The endometrial samples were fixed with 4% paraformaldehyde for 6–12 h at room temperature after removing blood and washed with phosphate-buffered saline (PBS). Then, all samples were processed into paraffin within 48 h; 4 μm-thicked formalin-fixed, paraffin-embedded endometrial tissue sections were deparaffinized with three successive passages through xylene and rehydrated through decreasing concentrations (100%, 95%, 80%, 70%, and 50%) of ethanol. The endogenous peroxidase activity was blocked by 3% hydrogen peroxide and the nonspecific binding was blocked by 5% bovine serum albumin (BSA) for 20 min. The sections were incubated with primary antibodies specific for PDK4 (1:200, Abgent, AP7041B), AR (1:200, Invitrogen, AN1-15), SIRT1 (1:200, cell signaling technology, 8469S) at 37 °C for 1 h, dissolved in 1% BSA (w/v in TBS) at the final concentration of 25 μg/mL. After three washes in 0.1% Tween 20 (v/v in PBS), sections were incubated for 30 min with secondary antibodies (Dako Cytomation) and washed again as before. Antibodies binding was detected with a brown precipitate after being stained with peroxidase substrate 3, 3-diaminobenzidine (DAB; Dako Cytomation) and counterstained with hematoxylin to allow visualization of the nuclei and dehydrated. For multiplex immunostaining, the endometrial tissue sections were rehydrated to proceed further with antigen retrieval, and blocked with the 5% BSA. Then the sections were incubated overnight with the following antibodies specific for PDK4 (1:100, Abgent, AP7041B), AR (1:100, Invitrogen, AN1-15), SIRT1 (1:200, cell signaling technology, 8469S). DAPI was used whenever sections were stained in fluorescence. The morphometric and fluorescence analysis was evaluated by the classical immunohistochemistry staining techniques [23]. Finally, the immunohistochemistry (IHC) and multiplex immunostaining sections were scanned by the Olympus research slide scanner VS200 (Olympus, Japan) and then quantified by the software Halo v3.3.2541.301 (Indica Labs, America). All images were scanned at × 20 magnification.

### Immunofluorescence

HESCs grown in 96-well cell culture plates, and endometrial tissue were washed with PBS and fixed with 4% paraformaldehyde (w/v) for 20 min at room temperature. After three 5-min washes with PBS, the fixed coverslips were permeabilized in PBS with 0.1% Triton X-100 for 5 min at room temperature. Nonspecific sites were blocked in PBS for 1 h at 37℃. The cells were incubated with fluorescein isothiocyanate-labeled phalloidin (1:200; P5282, Sigma-Aldrich) at 4 °C overnight. After washing with PBS three times, the cells were further incubated with Alexa Fluor 594-conjugated goat anti-rabbit IgG H&L (1:200, Abcam, ab150080), Alexa Fluor 594-conjugated goat anti-mouse IgG H&L (1:200, Abcam, ab150116), Alexa Fluor 647-conjugated goat anti-rabbit IgG H&L (1:200, Abcam, ab150079), Alexa Fluor 647-conjugated goat anti-mouse IgG H&L (1:200, Abcam, ab150115), and Alexa Fluor 488-conjugated goat anti-rabbit IgG H&L (1:200, Abcam, ab150077). Nuclei were stained with 4, 6-Diamidino-2-phenylindole dihydrochloride (DAPI) (Solarbio, China, C0065). Finally, images were captured by fluorescence confocal microscopy (PerkinElmer, USA). All images were scanned at × 20 magnification.

### Detection of IGFBP1 levels in cultured supernatants

IGFBP1 levels were measured using a Biotek Synergy H1/Synergy2 system with a Human IGFBP1 ELISA Kit (Abcam, UK, ab233618) according to the manufacturer’s instructions.

### Co-immunoprecipitation

The human endometrial stromal cells under in vitro decidualization for 3 days were used for co-immunoprecipitation. The decidual cells were lysed in whole-cell lysis buffer (50 mM Tris–HCl pH = 7.6, 150 mM NaCl, and 1.0% NP-40) containing a protease inhibitor cocktail. Next, 500 μg of each cell lysate was incubated with 1 μg antibody (anti-SIRT1) and was rotated slowly at 4 °C overnight. Then, the lysates were incubated with protein A/G PLUS-agarose beads (Abmart, Shanghai, China) at 4 °C for 2 h. The samples were washed in lysis buffer and resuspended in 10 μl 2 × fluorescent master mix (Proteinsimple, USA, SM-W004), heated at 95 °C for 10 min. All samples were analyzed by a western blot assay on the WES system to quantify the protein expression using an anti-SIRT1 antibody and an anti-PDK4 antibody.

### Animal

All animals were fed and treated at the Center for Animal Experiment of Wuhan University, and the animal anatomy procedures were conducted at the Animal Anatomy Laboratory of Shenzhen Zhongshan Obstetrics and Gynecology Hospital (formerly Shenzhen Zhongshan Urology Hospital). All animal protocols were approved by the Animal Care and Use Committee of Wuhan University (Ethical Approval Number: WDRY2019-K077). All the C57BL/6 J mice were purchased from the Guangdong Medical Laboratory Animal Center. C57BL/6 J mice (21 days) were fed in an independent ventilated germ-free cage at a constant temperature under a 12 h light:12 h darkness cycle. Female mice in estrus were mated with male mice or vasectomized male mice overnight in a 3:1 ratio.

The mice model of PCOS was developed by administering dehydroepiandrosterone (DHEA) (Aladdin, China, D106380) as described previously [24]. Briefly, female mice (25 days) were daily injected (subcutaneously) with DHEA (6 mg/100 g body weight, 100 μl/mouse in sesame oil (Aladdin, China, S304679) for 20 consecutive days. The vehicle control group was injected with 1 ml sesame oil daily for 20 consecutive days.

DHEA-treated female mice were mated with normal fertile or vasectomized male C57BL/6 J mice in the afternoon to induce pseudopregnancy. The vagina of female mice was examined the next morning at 8 a.m., and the morning following the appearance of a vaginal plug was defined as day 1 of pregnancy (D1) or day 1 of pseudopregnancy (PD1). After the mice were killed by cervical vertebra dislocation, the uterus of the mice was separated and stored at − 80 °C for real-time PCR and Western blot. The remaining uterine section was fixed in 4% paraformaldehyde for immunohistochemistry.

The model of artificially induced decidualization in mice was established as follows: PD4 female mice were anesthetized with 5% lidocaine, and 25 µL of corn oil was injected into the uterine horn on one side, while the other side was not treated as the control. Mice artificially induced decidualization were sacrificed in PD8.

### Uterine horn injection

It is reported that uterine horn injection of siRNA had an inhibited effect on implantation rate in mice [25]. To determine the effect of PDK4 on embryo implantation in vivo, si-PDK4 was injected into the uteri of mice. Briefly, siRNA was injected into one horn of the uteri on the morning of day 3 of pregnancy or pseudopregnancy and the other horn received scrambled control siRNA or water injection which served as control [26]. Then the mice’s uterine on day 7 of pregnancy was collected for observation.

### Statistical analysis

Statistical analyses were performed using SPSS 18.0 software (SPSS, USA). Graphing was performed using GraphPad Prism 7.0 software. Descriptive statistical analysis was performed on main clinical baseline characteristics and experimental data. Clinical values are expressed as median (interquartile range). Experimental data are expressed as mean ± standard deviation (SD). Comparisons were calculated using a t-test and adjusted by Bonferroni and Holm for multiplicity. More details for statistics are shown in each figure legend. *P* < 0.05 was considered statistically significant.

## Results

### Impaired decidualization in PCOS endometrial stromal cells

**Fig. 1:**
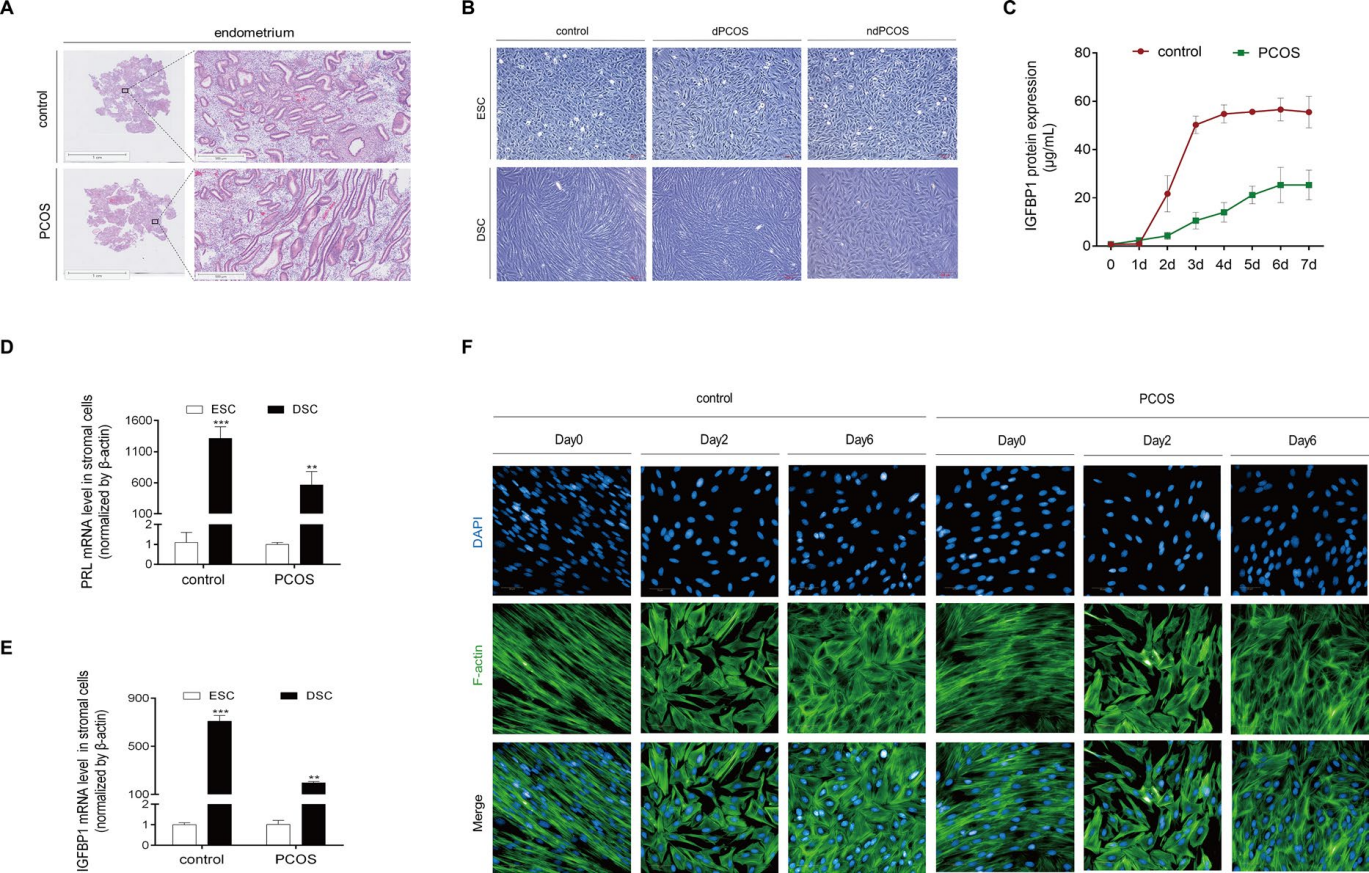
Impaired decidualization in PCOS endometrial stromal cells. **A** H&E staining analysis of endometrium in PCOS patients and control individuals. **B** Representative images displaying the morphology of endometrial stromal cells isolated from the control individuals and the PCOS individuals. The ‘control’ represents the decidualized controls, the ‘dPCOS’ represents the decidualized PCOS samples, and the ‘ndPCOS’ represents the PCOS samples failed to decidualize. The images of decidualized stomal cells were scanned at × 10 magnification. **C** Endometrial stromal cells from the control individuals and the PCOS individuals were cultured for 48 h, followed by treatment with 8-Br-cAMP and MPA for an additional 1–7 days. IGFBP1 that was released into the medium was measured using ELISA. Statistical significance is denoted by *p < 0.05, **p < 0.01, ***p < 0.001 when compared with endometrial stromal cells from the PCOS individuals (*n* = 3, one-way analysis of variance). **D** and **E** Endometrial stromal cells isolated from controls and PCOS patients were cultured for 48 h, followed by treatment with 8-Br-cAMP and MPA for additional 96 h, PRL (**D**) and IGFBP1 (**E**) mRNA levels were further measured by real-time PCR. Statistical significance is denoted by *p < 0.05, **p < 0.01, ***p < 0.001 when compared to endometrial stromal cells treated without 8-Br-cAMP and MPA. **F** Endometrial stromal cells isolated from controls and PCOS patients were cultured for 48 h, followed by treatment with 8-Br-cAMP and MPA for additional 2–6 days, and immunofluorescence staining analysis were further performed to analyze the morphological transformation, and the fluorescein isothiocyanate-labeled phalloidin was used to label actin filaments

The morphological characteristics of both the gland and stroma was determined in endometrial tissues (4 µm sections) by H&E staining following the manufacturer’s instructions. The endometrium of PCOS patients showed a synchronization of endometrial glands and poor response of stroma to sex hormone (Fig. 1A). All stromal cell cultures were monitored throughout the decidualization experiment for morphological changes, and a comprehensive dataset of images was systematically acquired from all cultures and treatments at day 7 prior harvesting. According to the morphological assessment, there were no observable morphological changes in the cultures without 8-Br-cAMP and MPA treatment on day 7 (Fig. 1B). However, within the subset of cultures treated with 8-Br-cAMP and MPA, it was observed that all 4 control samples and 5 PCOS samples underwent distinct morphological transition from a typical spindle-like stromal cells morphology to a cobblestone-shaped phenotype under the light microscope. Intriguingly, 3 PCOS samples retained an unaltered morphology, suggesting a potential compromise in the process of decidualization. The endometrial stromal cells from 4 controls (control) and 5 women with PCOS (dPCOS) successfully decidualized, while the endometrial stromal cells from 3 women with PCOS failed to decidualize (ndPCOS) after 7 days of treatment with 8-Br-cAMP and MPA (Fig. 1B). The clinical characteristics of PCOS individuals and controls, whose endometrium tissues were used for the morphology measurement, were shown in Supplemental Table 1. Decidualization was assessed by measuring secreted IGFBP1 protein levels within the conditioned media after 1–7 days of treatment with 8-Br-cAMP and MPA between the 4 control samples and 8 PCOS samples. The results showed a time-dependent increase in the levels of secreted IGFBP1 protein, with a peak on the 6th day after decidualization induction (Fig. 1C). The PCOS samples displaying aberrant decidualization morphology exhibited significantly lower IGFBP1 secretion peaks (28.24 ± 7.04 vs. 57.97 ± 4.73 μg/mL, *p* = 0.006) compared with controls (Fig. 1C). To validate the morphological findings and confirm the successful or impaired nature of decidualization process, the mRNA levels of PRL and IGFBP1 were also measured after 7 days of treatment with 8-Br-cAMP and MPA. The qRT-PCR data indicated that the PRL and IGFBP1 mRNA expression in response to 8-Br-cAMP and MPA in PCOS was significantly decreased (*P* < 0.01) compared with control samples (Fig. 1D, E). The treatment with 8-Br-cAMP and MPA induced a round and randomly arranged shape in control samples, while the PCOS samples showed a long fibroblast-like phenotype by using the fluorescein isothiocyanate-labeled phalloidin to label actin filaments (Fig. 1F). These data suggested that endometrial stromal cells derived from PCOS patients had impaired decidualization.

### Androgen excess impairs HESCs decidualization in vitro

**Fig. 2:**
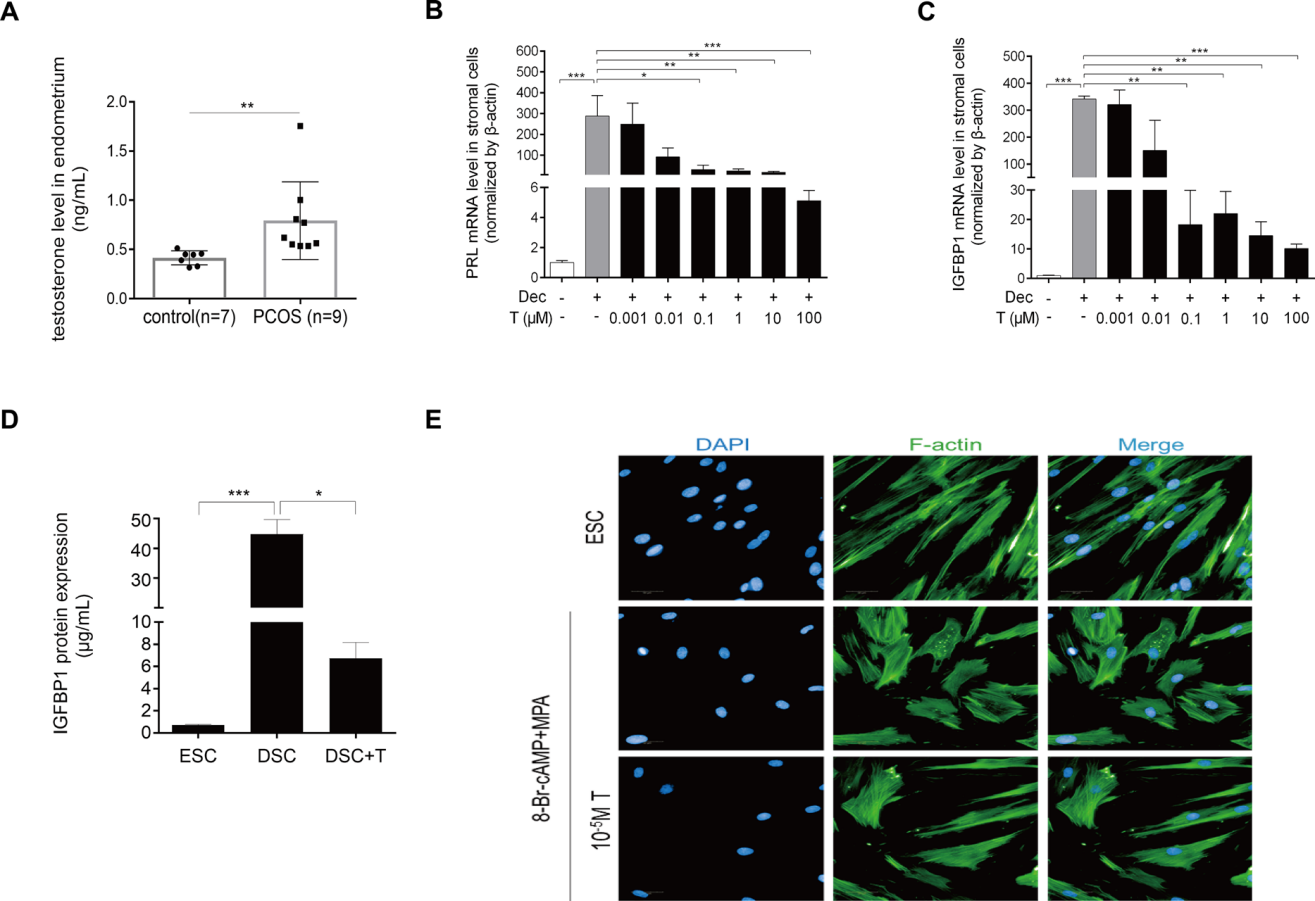
Changes of decidualization after testosterone treatment. **A** Testosterone levels in the endometrium of PCOS individuals and control individuals measured by ELISA. **B** and **C** Endometrial stromal cell lines (HESCs) were treated with 8-Br-cAMP and MPA for three days, followed by treatment with various concentrations of testosterone (0.001–100 μM) for an additional two days. PRL (**B**) and IGFBP1 (**C**) mRNA levels were further measured using real-time PCR. **D** and E HESCs were treated with 8-Br-cAMP and MPA for three days, followed by treatment with high concentration of testosterone (100 μM) for an additional two days. D IGFBP1 that was released into the medium treated with a high concentration of testosterone for 48 h was measured using ELISA. Statistical significance is denoted by *p < 0.05, **p < 0.01, ***p < 0.001. **E** Immunofluorescence staining analysis of HESCs to analyze the morphological transformation after being treated with 8-Br-cAMP and MPA or high concentration of testosterone, and the fluorescein isothiocyanate-labeled phalloidin was used to label actin filaments

The observation of impaired decidualization in endometrial stromal cells derived from PCOS patients prompted us to investigate the potential pathological mechanisms associated with endocrine disorders. Adverse effects of hyperandrogenism, insulin resistance, or chronic inflammation may serve as mechanistic approaches leading to the dysregulation of molecular or biochemical cascades requisite for endometrial receptivity in PCOS patients. These aberrations can impede endometrial growth, decidualization, and placentation, ultimately leading to pregnancy complications. In this study, we investigated the effects of hyperandrogenism on endometrium in PCOS patients. We found that the free testosterone levels within the endometrium tissue were significantly higher in the PCOS patients than that of the control group (Fig. 2A). These results suggested a remarkable role of excess testosterone in endometrial physiology. Furthermore, to investigate the effect of hyperandrogenic environment on endometrial decidualization, we pretreated the HESCs with 8-Br-cAMP and MPA to induce decidualization, and subsequently exposed the cells to testosterone to simulate the hyperandrogenic environment in the endometrium. Our findings revealed that elevated concentrations of testosterone exert a pronounced inhibitory effect on the mRNA levels of PRL and IGFBP1 within decidual cells (Fig. 2B, C). This inhibition was not confined to the transcript level, as androgen excess also significantly impede the production of decidual IGFBP1 protein (Fig. 2D). Furthermore, treatment with excess testosterone (10^−5^ M) induced a notable transition from a round and randomly arranged shape to a long fibroblast-like phenotype (Fig. 2E). These findings indicated an inhibitory effect of testosterone excess during decidualization.

### Patients characteristics and RNA-sequencing data analysis

Our previous study demonstrated that the endometrium of PCOS patients displays a distinct transcriptional profile and the dysregulation of endometrial transcriptional genes may be a cause of implantation failure [20]. Based on the previous study, we further analyzed the transcriptional profile of endometrium in PCOS with or without hyperandrogenism to identify the key target genes regulated by excess androgens. We performed an RNA-Seq analysis of the endometrium in PCOS patients stratified into two groups: those with hyperandrogenism (H_PCOS) and those without hyperandrogenism (N_PCOS). The clinical and biochemical characteristics of participants for transcriptome sequencing are listed in Table 1. As shown in Table 1, our analysis revealed that there were no statistically significant differences in terms of age, body mass index (BMI), basal follicle stimulating hormone (FSH), LH, thyroid stimulating hormone (TSH), fasting glucose, fasting insulin, or anti-Müllerian hormone (AMH) levels between the two groups (H_PCOS and N_PCOS), while the total T levels showed a substantial elevation in the H_PCOS group compared to the N_PCOS group.

**Fig. 3:**
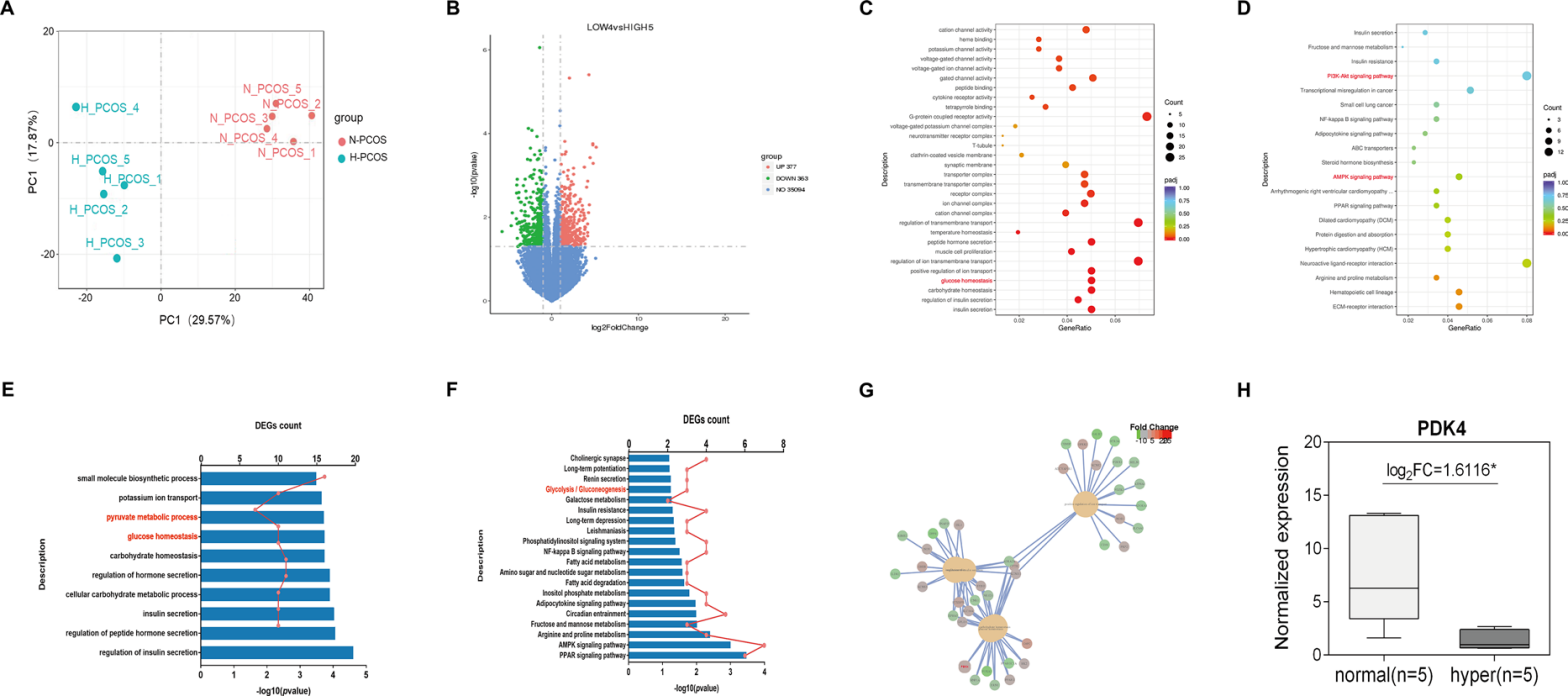
RNA-Seq results between PCOS patients with hyperandrogenism and without hyperandrogenism. **A** 2D scatter plot of the principal component analysis (PCA) results. Samples from the group of PCOS with hyperandrogenism (*n* = 5) and the group of PCOS without hyperandrogenism (*n* = 5) are represented as green dots and red dots, respectively. **B** Volcano plot of DEGs. Up- and downregulated DEGs with* p* < 0.01 and fold change > 2 are highlighted in light red and light green, respectively. **C** and **D** Statistical analysis of Gene Ontology (GO) functional classification (**C**) and KEGG pathway enrichment (**D**). The bubble size represents the count of genes annotated by the DEGs, and the color corresponds to the *p*-value. **E** GO functional classification of DEGs, highlighting the top-ranking terms associated with the pyruvate metabolic process.** F** KEGG pathway analysis of DEGs. The X-axis represents the number of DEGs (the number is presented as its square root value), and the Y-axis represents KEGG pathway terms. KEGG pathway terms related to glycolysis/gluconeogenesis are highlighted in red. **G** GO functional classification of downregulated DEGs. **H** RNA-Seq results for PDK4 expression in five PCOS patients with hyperandrogenism and five PCOS patients without hyperandrogenism

In general, the two significant clusters (the green and red dots) from principal component analysis (PCA) showed similar degrees of discrimination for different groups (Fig. 3A). A total of 740 DEGs were detected between the two groups, and 377 genes were upregulated in PCOS patients without hyperandrogenism, while 363 genes were highly expressed in PCOS patients with hyperandrogenism (Fig. 3B). GO analyses and KEGG pathway clustered these DEGs into distinct functional groups based on their involvement in biological processes, molecular functions and cellular components (Fig. 3C, D). Intriguingly, ‘Glucose homeostasis’ and ‘Glycolysis/Gluconeogenesis’ comprised the majority of the enriched GO and KEGG terms (Fig. 3E, F). In particular, the expression levels of PDK4, a representative enzyme that regulates the glycolysis process, was significantly decreased in the hyperandrogenism group compared with PCOS patients without hyperandrogenism (Fig. 3G). Through RNA-seq analysis, we found that PDK4 expression was significantly downregulated in the endometrium of PCOS women with hyperandrogenism (Fig. 3H), while other members of the PDK family displayed no significant changes (Supplemental Fig. S1A–C).

### PDK4 induction under decidualization and silence of PDK4 results in abnormal decidualization both in vivo and in vitro

**Fig. 4:**
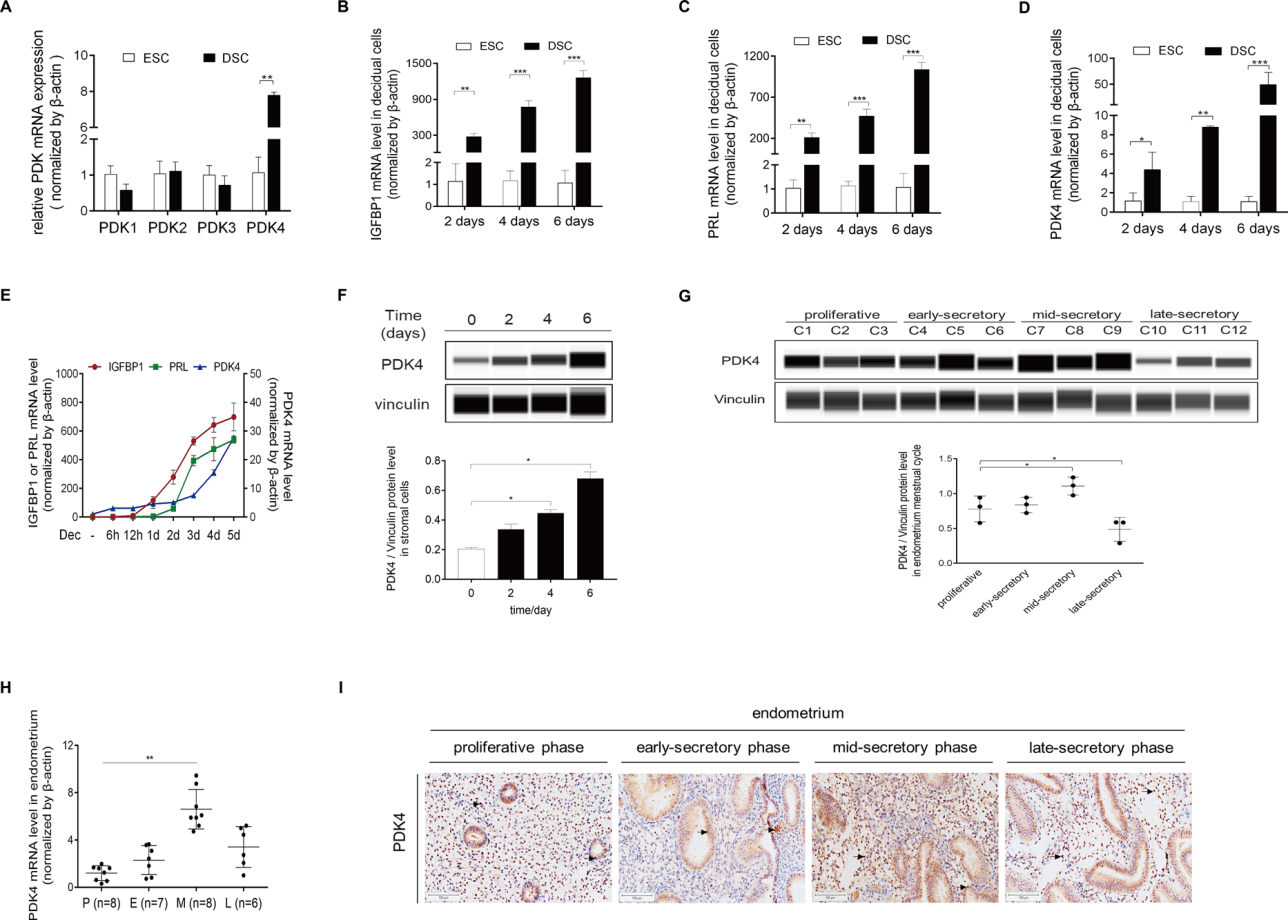
PDK4 induction during decidualization. **A** Real-time PCR analysis of PDK1, PDK2, PDK3, and PDK4 in HESCs treated with 8-Br-cAMP and MPA for three days.** B**,** C,** and** D** HESCs were cultured for 48 h, followed by treatment with 8-Br-cAMP and MPA for an additional two, four, and six days. IGFBP1 (**B**), PRL (**C**), and PDK4 (**D**) mRNA levels were further measured using real-time PCR. Statistical significance is denoted by *p < 0.05, **p < 0.01, ***p < 0.001, comparing treated samples with those without 8-Br-cAMP and MPA treatment. **E** Correlations analysis between PDK4 and IGFBP1 or PRL mRNA expression in HESCs during decidualization. **F** Western blot analysis of PDK4 protein during decidualization. **G** Western blot analysis of PDK4 protein in the endometrium throughout the menstrual cycle. Statistical significance is denoted by *p < 0.05 compared with endometrium from the proliferative phase. **H** Real-time PCR analysis of PDK4 mRNA levels in the endometrium throughout the menstrual cycle. **I** Immunohistochemistry staining analysis of PDK4 protein distribution in human endometrium during the menstrual cycle

The above-described findings encouraged us to further investigate the role of PDK4 in decidualization. In murine model, there exists an induction of genes associated with glycolysis and a notable increase in lactate production within decidua, suggesting the activation of Warburg-like glycolysis. However, it remains unknown whether Warburg-like glycolysis is active during human decidualization. PDK4 compromises the activity of Pyruvate Dehydrogenase Complex (PDC) through the phosphorylation of Pyruvate Dehydrogenase E1 Alpha (PDHE1α), thereby attenuating pyruvate oxidation and promoting pyruvate dehydrogenation. Among the four isoforms of PDK that can phosphorylate PDHE1α, it was observed that, unlike PDK1, PDK2, and PDK3, the expression of PDK4 was significantly increased under in vitro decidualization (Fig. 4A), suggesting that PDK4 may serve as the predominant form during decidualization. PDK4 mRNA expression displayed a time-dependent increase in stromal cells undergoing in vitro decidualization over periods of 2, 4, and 6 days, mirroring the temporal pattern observed for IGFBP1 and PRL (Fig. 4B–E). Complementary western blot assay corroborated the upregulation of PDK4 protein levels under in vitro decidualization (Fig. 4F). In the endometrium of control individuals, the protein and mRNA levels of PDK4 remained relatively stable during the proliferative phase and early-secretory phase, but notably increased during the mid-secretory phase, followed by a remarkable decrease in the late-secretory phase in stromal cells (Fig. 4G, H). Immunohistochemistry staining for PDK4 revealed its presence in both glandular epithelium and stromal cells during the menstrual cycle, with a significant increase in stromal cells during the mid-secretory phase (Fig. 4I). These observations collectively suggest a dynamic and context-dependent regulation of PDK4 expression in the endometrium, underscoring its potential relevance during decidualization.

**Fig. 5:**
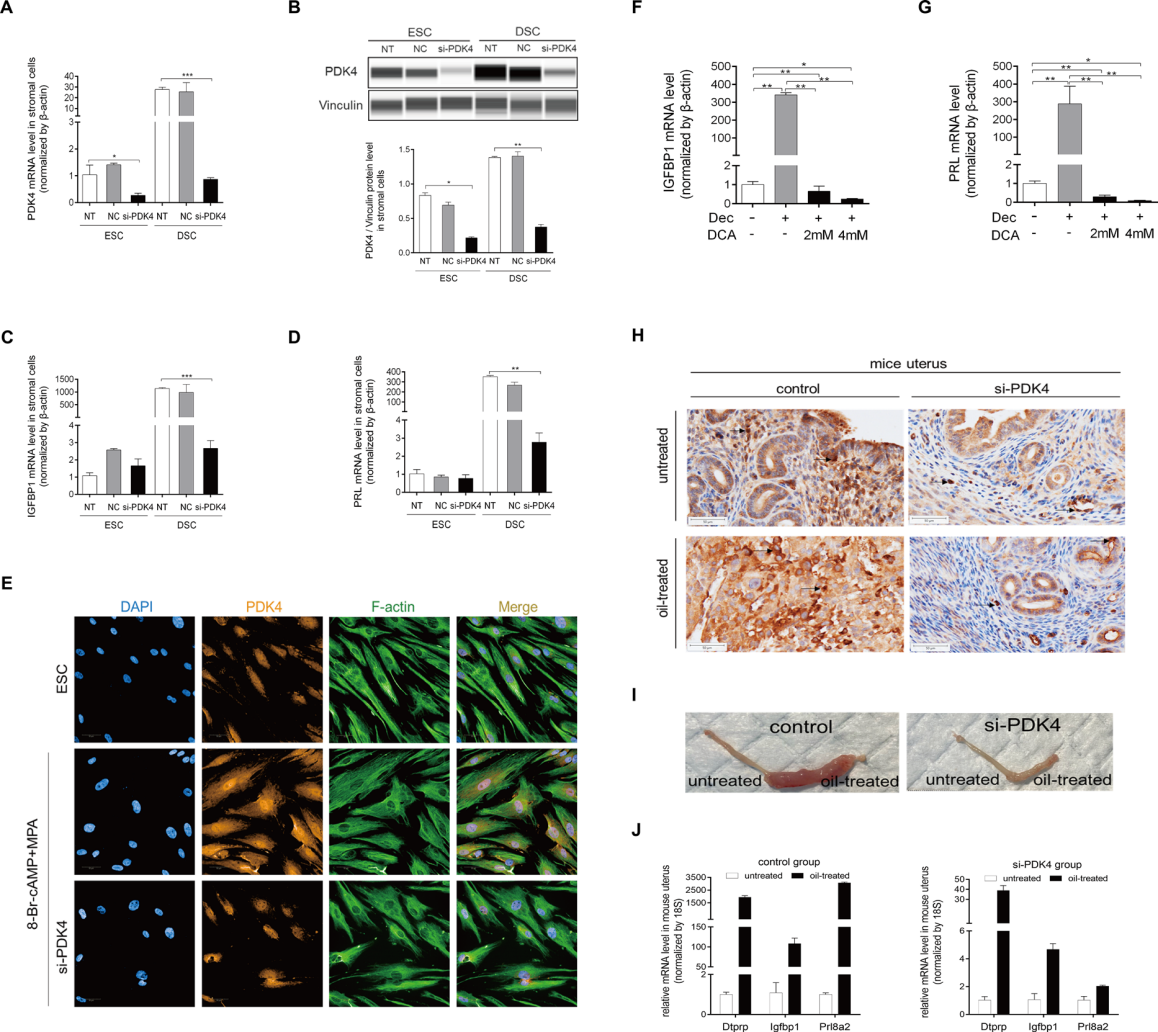
PDK4 function on decidualization. **A** PDK4 mRNA level in endometrial stromal cells (ESCs) and decidual stromal cells (DSCs) transfected with control siRNA (NC) or si-PDK4.** B** PDK4 protein levels in ESCs and DSCs transfected with control siRNA (NC) or si-PDK4. **C** PRL mRNA level after transfection with PDK4 siRNA. **D** IGFBP1 mRNA level after transfection with PDK4 siRNA. Statistical significance is denoted by *p < 0.05, **p < 0.01, ***p < 0.001 compared with untreated HESCs. **E** Immunofluorescence analysis of the morphological transformation of HESCs after transfection with PDK4 siRNA. The fluorescein isothiocyanate-labeled phalloidin was used to label actin filaments. **F** Real-time PCR analysis of the effects of DCA on PRL mRNA level. **G** Real-time PCR analysis of the effects of DCA on IGFBP1 mRNA level. Statistical significance is denoted by *p < 0.05, **p < 0.01, ***p < 0.001. **H** Immunochemistry staining analysis of PDK4 expression in the control side and the oil-injected side of the uterus in the control group and the si-PDK4 group. **I** Images showing the gross morphology of the uterus. The stimulated uterine horns formed robust deciduoma, while the si-PDK4 treated uterine horns showed an impaired deciduoma. **J** mRNA expression of Dtprp, Igfbp1, and Prl8a2 was significantly increased on the stimulated horn of the uterus in the control group, and the induction was abrogated by the treatment of PDK4 siRNA injection. Statistical significance is denoted by *p < 0.05, **p < 0.01, ***p < 0.001 compared with the untreated uterine horn

To further investigate the function of PDK4 on decidualization, siRNA was used to knock down PDK4 in HESCs. Both PDK4 mRNA and protein levels were significantly reduced by the application of si-PDK4 during decidualization (Fig. 5A, B). Subsequently, PRL and IGFBP1 mRNA levels were also remarkably suppressed by PDK4 knockdown (Fig. 5C, D). Furthermore, decidualized HESCs displayed polygonal cell morphologies with a random distribution of F-actin filaments compared with non-decidualized HESCs. The knockdown of PDK4 induced a noticeable transformation from a round and randomly arranged shape to a long fibroblast-like phenotype (Fig. 5E). To further elucidate the functional significance of PDK4 during decidualization, we employed Dichloroacetate (DCA), an inhibitor known to target all four PDK isoforms. Given that PDK4 emerged as the dominant isoform during decidualization in our study, it was expected that DCA would primarily affect PDK4 activity. Indeed, under in vitro decidualization, PRL and IGFBP1 mRNA levels were significantly inhibited by DCA treatment (Fig. 5F, G). Decidualization can be induced naturally by blastocysts implantations, or by the intraluminal infusion of oil in pseudopregnancy mice. In this regard, we established an in vivo decidualization model wherein sesame oil was injected into one horn of uteri on day 4 of pseudopregnancy, whereas the other horn remained untreated as the control. On day 8 of pseudopregnancy, the uterine samples from mice model were collected for analysis. Immunohistochemistry staining revealed that PDK4 was primarily highly expressed in decidual cells within the oil-treated horn of the uterine (Fig. 5H). Furthermore, the significantly increased wet weight and decidual/trophoblast prolactin-related protein (Dtprp), Igfbp1, and prolactin family 8, subfamily a, member 2 (Prl8a2) mRNA levels in the oil-treated horn of the uterine suggested that the artificial decidualization model in vivo was successfully established (Fig. 5I, J). To further identify the regulatory role of PDK4 in decidualization, we utilized siRNA to knock down the expression of PDK4 in pseudopregnancy mice uterus. The results demonstrated that si-PDK4 treatment significantly downregulated PDK4 expression compared with the untreated horn (Fig. 5H). Importantly, this intervention also resulted in a remarkable decrease in the wet weight and Dtprp mRNA levels, providing compelling evidence of PDK4’s involvement in decidualization in an in vivo setting (Fig. 5I, J).

### The expression of PDK4 is aberrantly decreased in PCOS patients

**Fig. 6:**
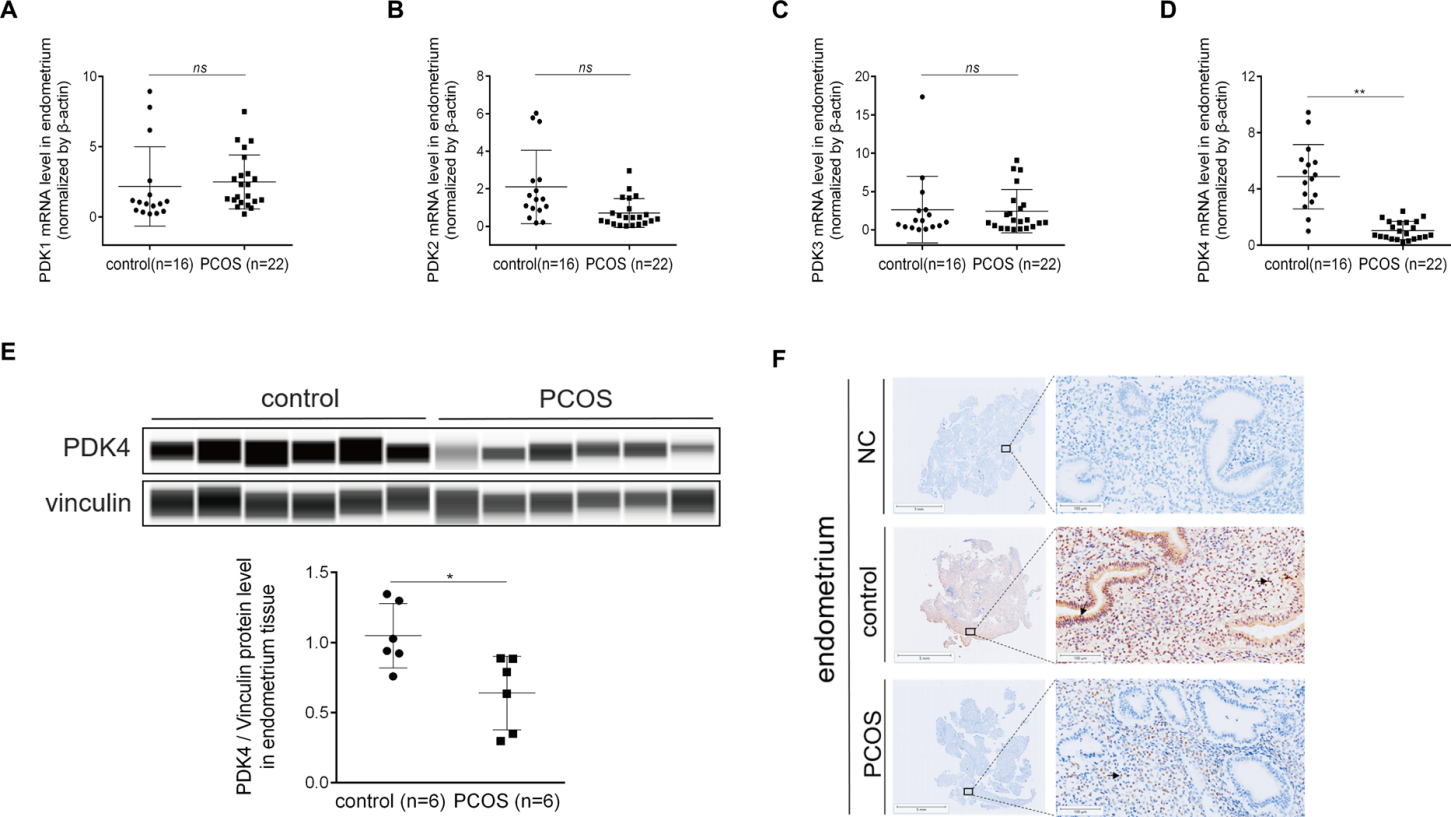
Aberrant expression of PDK4 in the endometrium of PCOS patients. The mRNA expression levels of PDK1 (**A**), PDK2 (**B**), PDK3 (**C**), and PDK4 (**D**) in the endometrium of PCOS patients. **E** Total PDK4 protein levels normalized to total Vinculin protein levels in Western blot analysis. **F** Immunohistochemistry staining analysis of PDK4 in the endometrium of PCOS patients. Statistical significance is denoted by *p < 0.05, **p < 0.01

In line with our prior investigation, wherein we elucidated the distinct transcriptional profile exhibited by the endometrium of PCOS patients and its potential association with impaired decidualization as a contributor to implantation failure, we conducted an in-depth analysis of PDK4 expression within this context. Our RNA-seq analysis revealed that PDK4 expression was significantly downregulated in the endometrium of PCOS patients, while no discernible differences were observed in PDK1, PDK2, and PDK3 expression (Fig. 6A–D). Immunohistochemistry staining analysis showed that significantly lower expression of PDK4 was detected in the PCOS group compared with the control group (Fig. 6E). This aberrant expression pattern was further validated through western blot analysis, which similarly indicated a decreased endometrial PDK4 level in the PCOS group compared with the control group (Fig. 6F). These observations collectively underscore the perturbed expression of PDK4 within the stromal cells of PCOS patients and highlight the potential role of PDK4 in the decidualization, a pivotal step that renders the uterine environment receptive to embryo implantation.

### Androgen excess downregulates PDK4 to inhibit stromal decidualization

**Fig. 7:**
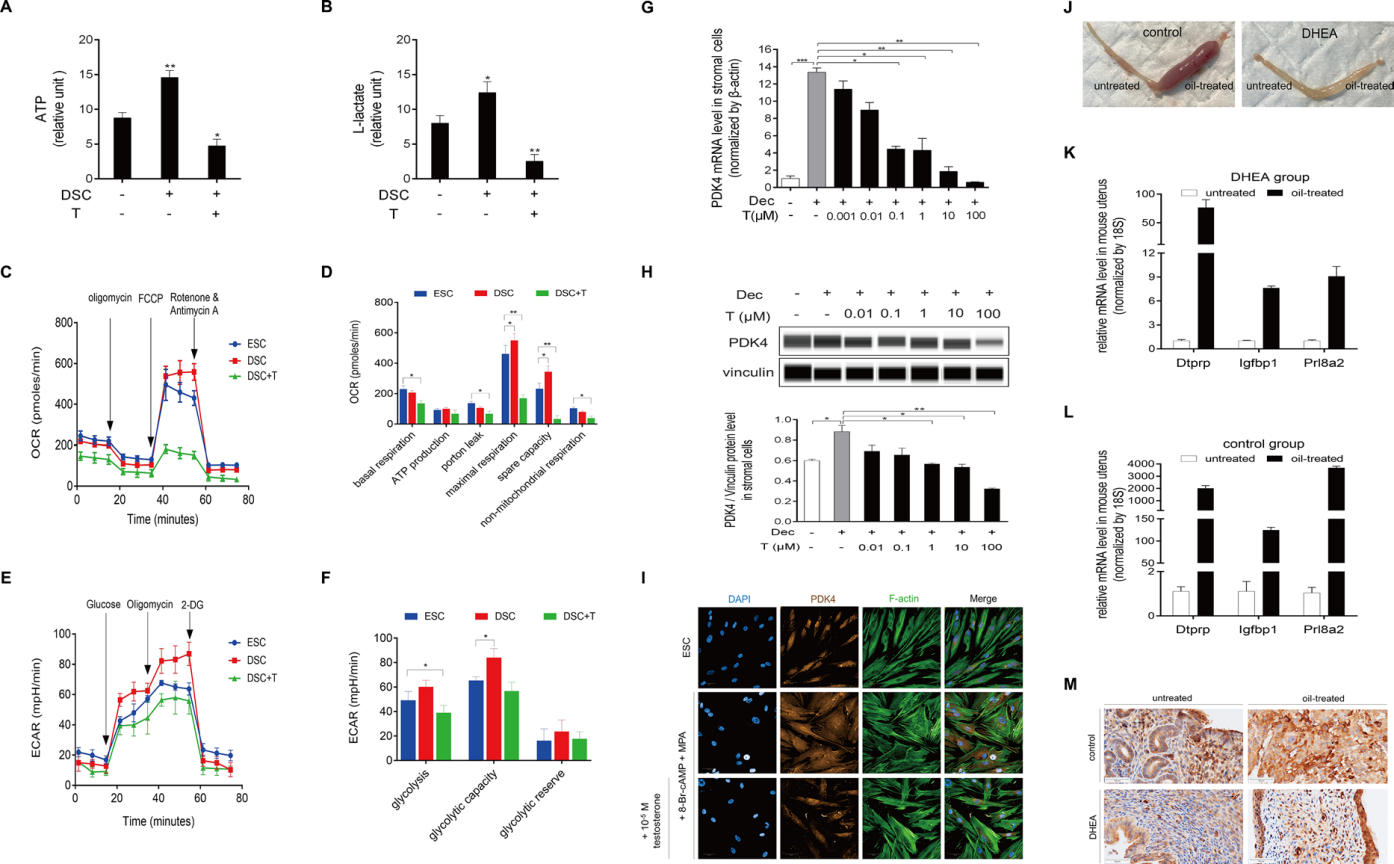
Androgen excess inhibits glycolysis and PDK4 expression in decidual cells. **A** and **B** HESCs were pretreated with 8-Br-cAMP and MPA for three days, followed by treatment with a high concentration of testosterone (100 μM) for an additional two days. ATP production and L-lactate production were further measured using a spectrophotometer. Statistical significance is denoted by *p < 0.05, **p < 0.01 compared with untreated HESCs. **C** Seahorse XFp assays were employed to measure the oxygen consumption rate (OCR) in HESCs treated with 8-Br-cAMP, MPA and a high concentration of testosterone (100 μM). **D** Various mitochondrial respiration parameters, including basal respiration, ATP production, maximal respiration, spare respiratory capacity, proton leak, and non-mitochondrial respiration, were assessed after being treated with a high concentration of testosterone (100 μM). Statistical significance is denoted by *p < 0.05, **p < 0.01 compared with untreated HESCs. **E** The Seahorse XFp assay detected the extracellular acidification rate (ECAR) in HESCs treated with 8-Br-cAMP, MPA and a high concentration of testosterone (100 μM). **F** Quantification of glycolysis, glycolytic capacity, and glycolytic reserve after treatment with 8-Br-cAMP, MPA and a high concentration of testosterone (100 μM). **G** HESCs were treated with 8-Br-cAMP and MPA for three days, followed by treatment with various concentrations of testosterone (0.001–100 μM) for an additional two days. PDK4 mRNA levels were further measured by real-time PCR. Statistical significance is denoted by *p < 0.05, **p < 0.01, and ***p < 0.001. **H** PDK4 protein levels in HESCs after being treated with 8-Br-cAMP, MPA or testosterone. Statistical significance is denoted by *p < 0.05, **p < 0.01. **I** Immunofluorescence staining analysis of HESCs to analyze the morphological transformation after being treated with 8-Br-cAMP and MPA or high concentration of testosterone (100 μM), and the fluorescein isothiocyanate-labeled phalloidin was used to label actin filaments, and Alexa Fluro 594 dye was used to label PDK4. **J** Images showing the gross morphology of the uterus in the control group, where stimulated uterine horns formed robust deciduoma, and in the DHEA-induced group, where stimulated uterine horns showed impaired deciduoma. **K** and** L** mRNA expression of Dtprp, Igfbp1, and Prl8a2 were significantly increased on the stimulated horn of the uterus in the control group (**K**), and the induction was abrogated by the treatment of DHEA injection (**L**). Statistical significance is denoted by *p < 0.05, **p < 0.01, ***p < 0.001 compared with the uterine corn without any treatment. **M** Immunohistochemistry staining analysis of PDK4 in the uterus from the control and DHEA-induced mice. The black arrow indicates a positive signal

To further elucidate the mechanism of impaired decidualization in PCOS patients, we first analyzed the change in glucose metabolism after excess testosterone treatment. Interestingly, we found that excess testosterone significantly inhibits ATP production and lactate production (Fig. 7A, B). Cellular bioenergetics profile analysis using a Seahorse XFp analyzer revealed that androgen excess in decidual cells led to a decrease in mitochondrial basal respiration and proton leakage, and the difference between maximal respiration rate and spare respiratory capacity was statistically significant (*p* < 0.05) (Fig. 7C, D). Furthermore, we explored the effect of androgen excess on glycolysis. The results of the extracellular acidification rate (ECAR) assay showed that glycolysis displayed a decrease after being treated with high concentrations of testosterone and decidual stromal cells showed an increase in glycolytic capacity (Fig. 7E, F). Under in vitro decidualization, glycolysis-related genes (GLUT1, glucose-6-phosphate dehydrogenase (G6PD), p-PDHE1α, and lactate dehydrogenase A (LDHA)) were also significantly elevated (Fig. S2A). p-PDHE1α, a subunit of PDC, can oxidatively decarboxylate pyruvate and act as a surrogate marker of PDK activity [27]. PDK4 can inactivate PDC through phosphorylating PDHE1α, and silence of PDHE1α can compromise PDC activity. Knockdown of PDHE1α also led to the downregulation of PRL and IGFBP1 mRNA levels (Fig. S2C, D). Moreover, the PDK4 mRNA and protein levels were decreased by testosterone excess (Fig. 7G, H). This hormonal perturbation also induced a long fibroblast-like phenotype of decidual stromal cells, and downregulated the protein level of PDK4 (Fig. 7I). To further ascertain the impact of androgen excess in decidualization in vivo, we then established a PCOS-like mice model through subcutaneous injection of DHEA for 21 days following the previous protocols [28]. The results showed that the PCOS-like mice exhibited increased body weight, disordered estrous cycles, follicular-like ovaries, and hypertrophic adipose tissue (Supplemental Fig. S3A–D). Interestingly, the decidualization of these PCOS-like mice uterus was significantly damaged (Fig. 7J). As shown in the results, the wet weight and Dtprp mRNA levels were remarkably reduced in the DHEA-induced group. (Fig. 7K, L). Immunohistochemistry staining was employed to determine the PDK4 expression in mice uterus during decidualization. The results showed an increase in PDK4 expression induced by decidualization, while the induction was inhibited by androgen excess treatment (Fig. 7M). These data suggested that PDK4 is a pivotal target of androgen excess during decidualization, and that androgen excess may downregulate PDK4 as part of its mechanism to inhibit decidualization.

### SIRT1 acts at the upstream of PDK4 in decidual cells

**Fig. 8:**
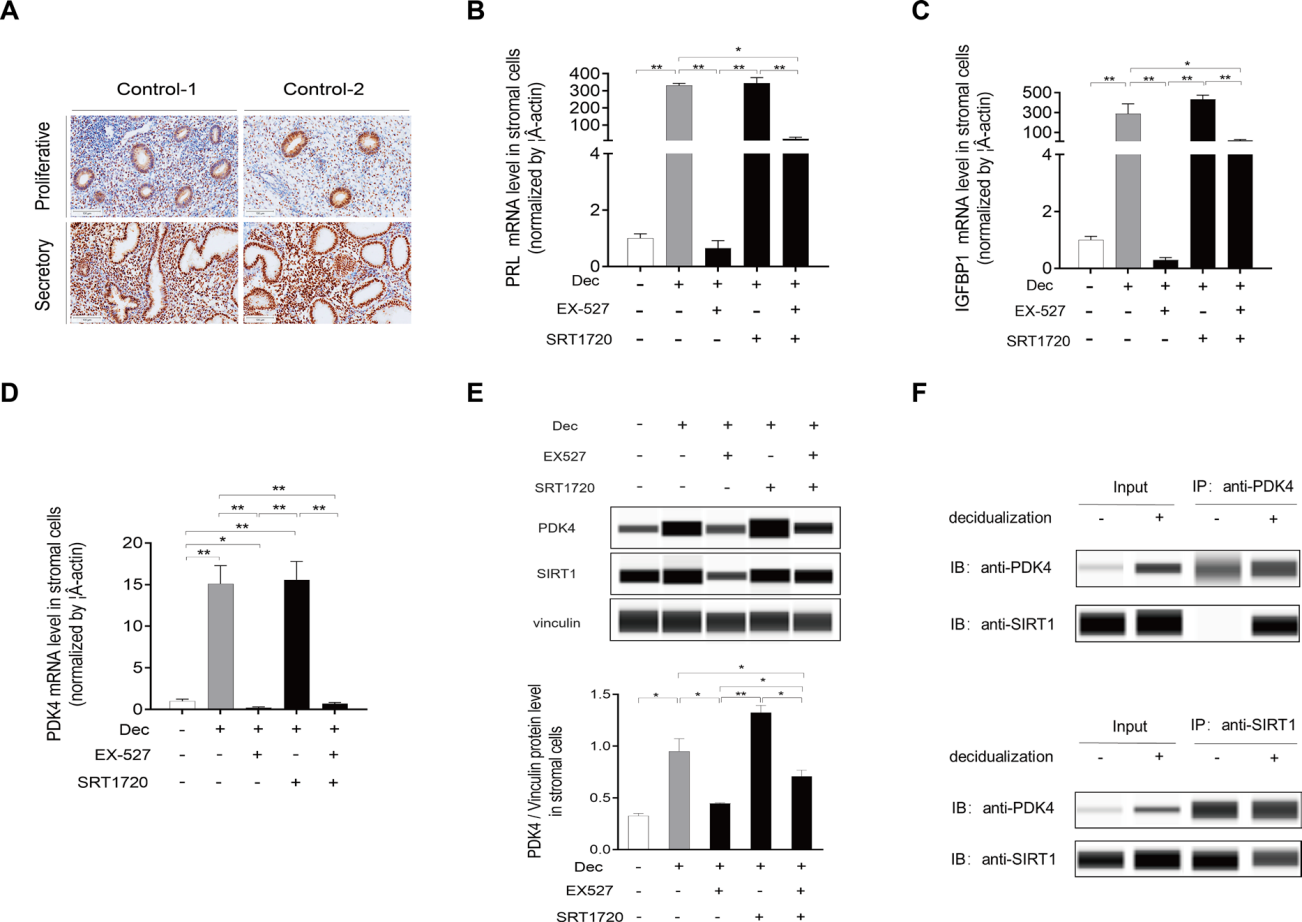
SIRT1 acts as the upstream of PDK4. **A** Immunohistochemistry staining analysis of SIRT1 in the endometrium during the menstrual cycle. **B**, **C**, and** D** HESCs were pretreated with 8-Br-cAMP and MPA for three days, followed by treatment with EX-527 (1 nM) and SRT1720 (5 nM) for an additional two days. PRL (**B**), IGFBP1 (**C**), and PDK4 (**D**) mRNA levels were further measured using real-time PCR. Statistical significance is denoted by *p < 0.05, **p < 0.01. **E** PDK4 and SIRT1 protein levels were measured using Western blot analysis in HESCs. **F** Proteins were extracted from HESCs treated with or without 8-Br-cAMP and MPA for three days. Immunoprecipitation was performed using an anti-PDK4 antibody or an anti-SIRT1 antibody, followed by Western blot analysis to investigate the interaction between PDK4 and SIRT1

SIRT1 is the most well-studied member of Sirtuins family and acts as a regulator of forkhead box O1 (FOXO1) in islet function [29]. Given the critical role of FOXO1 in endometrial receptivity, we posited that SIRT1 might be a principal regulator governing the process of decidualization. As a deacetylase, SIRT1 plays an important role in multiple physiological processes within the endometrium, including angiogenesis, oxidative stress response, and modulation of the immune microenvironment [30–32]. Recently, Magdalina et al. corroborated the significant role of SIRT1 in endometrial decidualization using Sirt1 uterus specific knockout mice [33]. However, the precise mechanism by which SIRT1 regulates the process of decidualization remains elusive. To investigate the temporal expression patterns of SIRT1, we conducted immunohistochemistry staining, revealing a significant upregulation of SIRT1 during the secretory phase (Fig. 8A). After being treated with SRT1720, a SIRT1 activator, PRL and IGFBP1 mRNA expression levels in decidual cells were markedly increased compared with non-decidualized cells, but not compared with decidualized cells. Furthermore, the expression of PRL and IGFBP1 was significantly decreased in decidual cells treated with EX527, a pharmacological agent renowned for its capacity to inhibit the deacetylase activity of SIRT1, compared with decidualized cells (Fig. 8B, C). Furthermore, the expression of PRL and IGFBP1 was significantly decreased in decidual cells treated with EX527 compared with decidualized cells (Fig. 8B, C). These results suggested that the inhibition of SIRT1 may lead to an impaired decidualization. To further investigate the influence of SIRT1 on PDK4 function during decidualization, the PDK4 levels were determined. It was shown that both the PDK4 mRNA and protein levels exhibited significant stimulation in response to SRT1720 treatment, whereas they were significantly down-regulated by EX527 treatment (Fig. 8D, E). Co-immunoprecipitation was used to examine the interaction between SIRT1 and PDK4 during decidualization. These experiments yielded confirmation of a physical interaction between SIRT1 and PDK4, as evidenced by the immunoprecipitation of SIRT1 by the PDK4 antibody in decidual stromal cells (Fig. 8F), implying the potential role of SIRT1 in governing PDK4 function during decidualization.

### Androgen excess inhibits SIRT1/PDK4 signal through AMPK

**Fig. 9:**
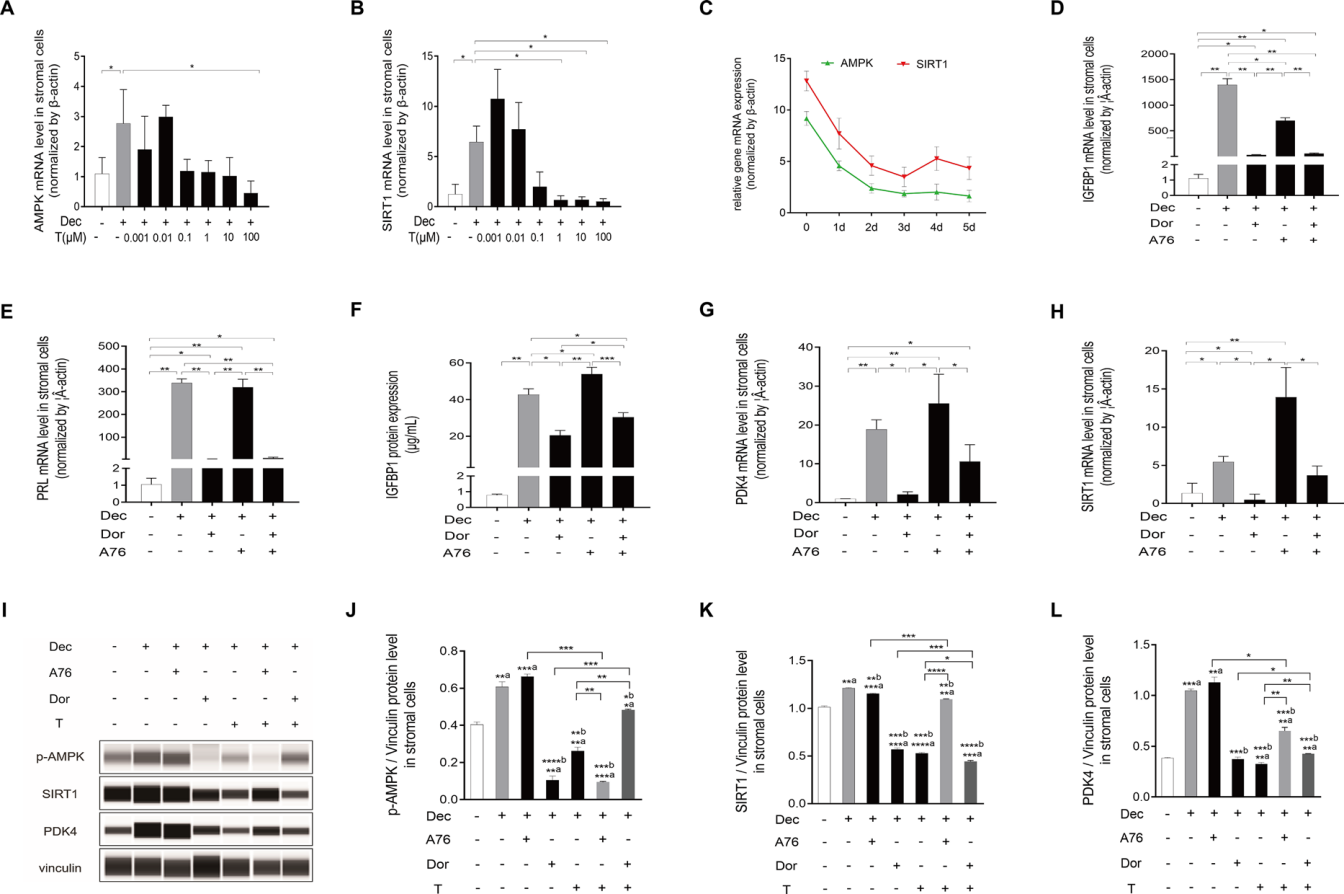
Effects of AMPK on decidualization. **A** and **B** HESCs were pretreated with 8-Br-cAMP and MPA for three days, followed by treatment with various concentrations of testosterone (0.001–100 μM) for an additional two days. AMPK (**A**) and SIRT1 (**B**) mRNA levels were further measured using real-time PCR. Statistical significance is denoted by *p < 0.05. **C** HESCs were treated with 8-Br-cAMP and MPA for 1–5 days, and AMPK and SIRT1 mRNA levels were further measured using real-time PCR. **D**-I HESCs were pretreated with 8-Br-cAMP and MPA for three days, followed by treatment with Dorsomorphin (5 μM) or A-769662 (20 μM) alone or combining Dorsomorphin/A-769662 with testosterone (100 μM) for an additional two days. IGFBP1 (**D**) and PRL (**E**) mRNA levels were further measured using real-time PCR. IGFBP1 protein (**F**) that was released into the medium was measured using ELISA. PDK4 (**G**) and SIRT1 (**H**) mRNA levels were further measured using real-time PCR. Statistical significance is denoted by *p < 0.05, **p < 0.01. I Proteins of p-AMPK, SIRT1, and PDK4 were measured using Western blot analysis. Quantitative analysis of p-AMPK (J), SIRT1 (K), and PDK4 (L) protein expression levels were showed in histogram. **a** represents the treated cells vs. non-decidualized cells. **b** represents the treated cells vs. decidualized cells without other treatments. Statistical significance is denoted by *p < 0.05, **p < 0.01, ***p < 0.001, ****p < 0.0001

AMPK acts as a regulator of PDK4 in muscle and cardiomyocytes. However, it is unknown whether AMPK is essential for the induction of PDK4 during decidualization. To provide some clarity, we performed a series of experiments involving varying dose- and time-courses of testosterone exposure. We found that AMPK and SIRT1 mRNA expression were downregulated by testosterone excess (Fig. 9A, B), and the inhibition showed a time-dependent trend (Fig. 9C). To further elucidate the role of AMPK in regulating the decidualization process, we treated stromal cells with A76 (20 μM), a p-AMPK activator that displays selectivity towards β1 subunit-containing heterotrimers. Compared with the non-decidualized cells, the treatment with A76 resulted in a marked increase in IGFBP1 and PRL mRNA expression, and Dorsomorphin (5 μM), a p-AMPK inhibitor, also slightly stimulated the expression of IGFBP1 and PRL. However, the induction of IGFBP1 and PRL by A76 was abrogated by Dorsomorphin (Fig. 9D, E). IGFBP1 protein levels were significantly increased by A76 compared with the decidualized cells (Fig. 9F). Both qRT-PCR and western blot analysis were performed to validate the regulatory impact of AMPK on SIRT1/PDK4 pathway. A76 stimulation elicited a modest increase in PDK4 mRNA and protein levels, whereas Dorsomorphin treatment notably suppressed these levels compared with decidualization alone (Fig. 9G, L). Notably, the augmented PDK4 expression induced by A76 was attenuated by Dorsomorphin, indicating a regulatory influence of p-AMPK on PDK4 (Fig. 9G). Furthermore, Dorsomorphin treatment significantly downregulated both mRNA and protein levels of SIRT1 compared with the decidualized cells, suggesting a regulatory role of p-AMPK on SIRT1 expression (Fig. 9H, K). The western blot analysis results further demonstrated that the expression levels of p-AMPK were decreased by Dorsomorphin and high concentrations of testosterone, while SIRT1 and PDK4 protein were also downregulated by high concentrations of testosterone (Fig. 9J–L). Furthermore, the inhibition of SIRT1 and PDK4 expression by high concentrations of testosterone was abrogated by A76 (Fig. 9K, L). Collectively, our study provides insights into the regulatory mechanisms underlying androgen excess in human decidualization, with a proposed schematic pathway depicting how androgen excess regulates this process via AMPK/SIRT1/PDK4 signaling pathway.

### Androgen receptor (AR) is essential for androgen function during decidualization

**Fig. 10:**
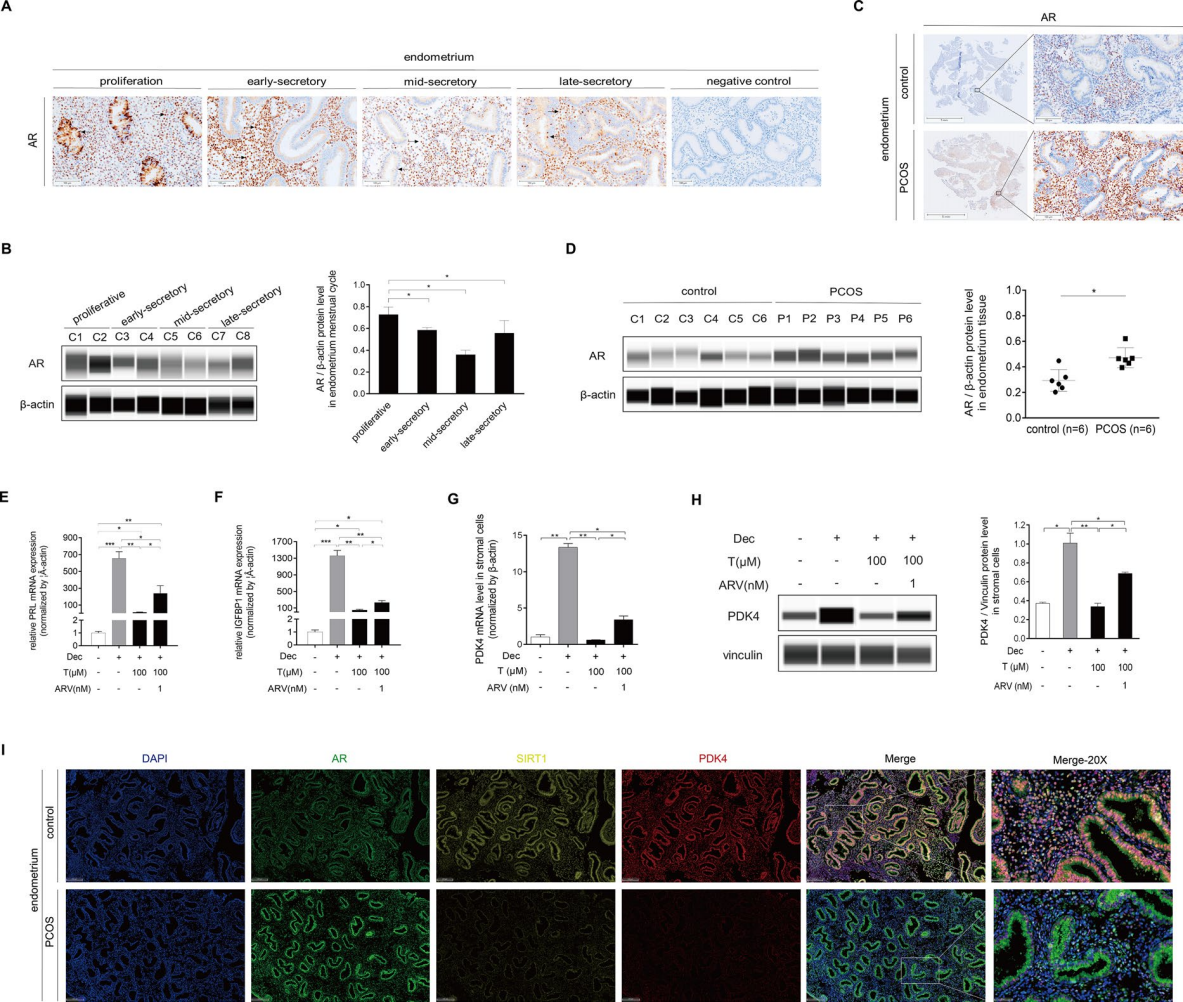
The androgen receptor is essential for androgen function during decidualization. **A** Immunohistochemistry staining analysis of AR in human endometrium during the menstrual cycle. **B** Western blot analysis of AR in human endometrium during the menstrual cycle. **C** Immunohistochemistry staining analysis of AR in the endometrium of the control individuals and PCOS patients. **D** Western blot analysis of AR protein levels in the endometrium of the control individuals and PCOS patients. E–H HESCs were treated with 8-Br-cAMP and MPA for three days, followed by treatment with testosterone (100 μM) and ARV-110 (1 nM) for an additional two days. PRL (**E**), IGFBP1 (**F**), and PDK4 (**G**) mRNA levels were further measured using real-time PCR, and PDK4 protein levels (H) were further measured using a western blot analysis. Statistical significance is denoted by *p < 0.05, **p < 0.01. **I** Multiple fluorescence immunohistochemistry staining analysis of endometrium in the control individuals and PCOS patients

**Fig. 11:**
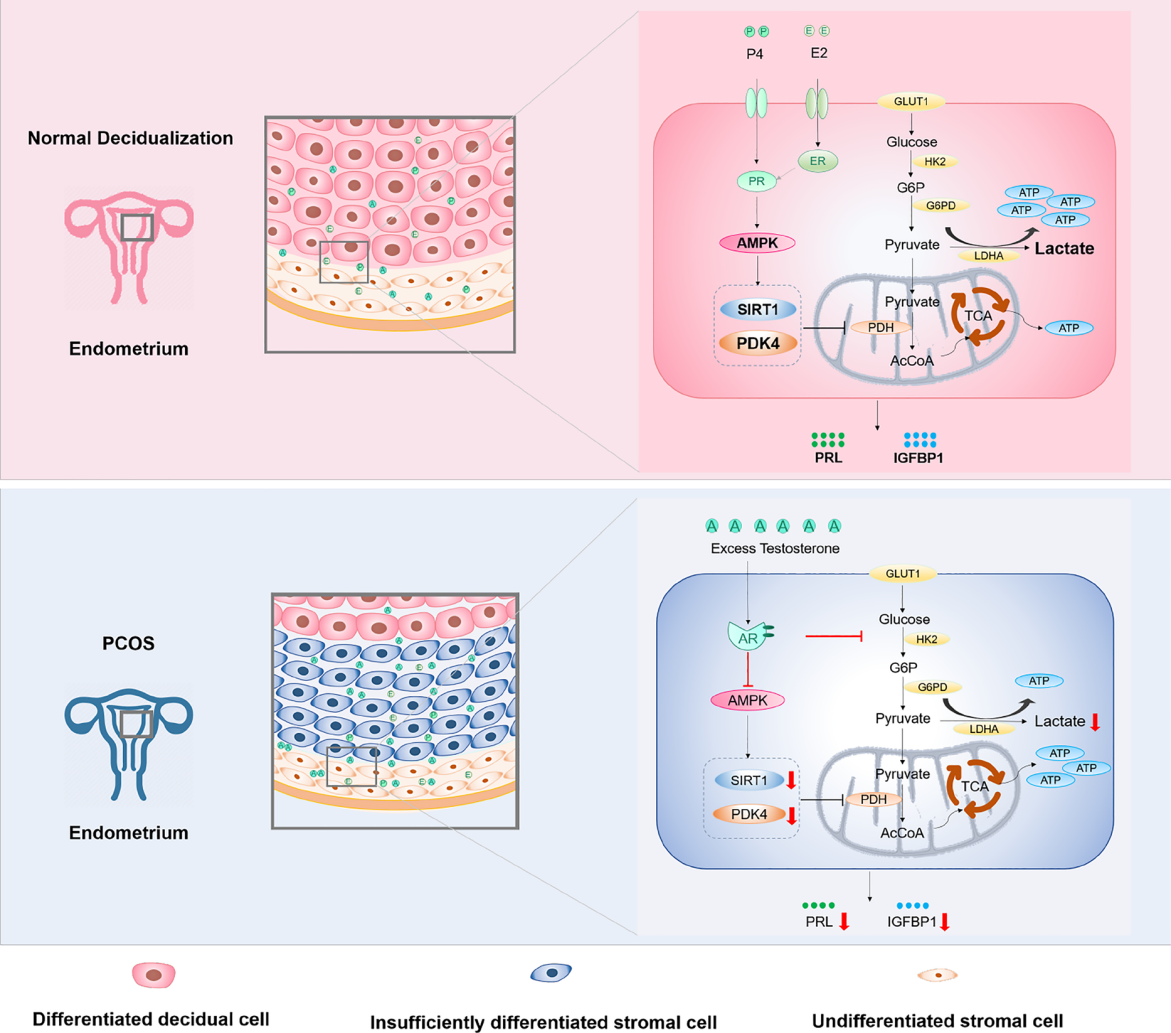
Androgen excess regulates decidualization via the AR/AMPK/SIRT1/PDK4 pathway. PDK4 levels were induced during decidualization. PDK4 expression decreased in the endometrium of PCOS patients. Androgen excess regulates decidualization via the AR/AMPK/SIRT1/PDK4 pathway

AR is a ligand-activated transcription factor in both the male and female reproductive tracts whose activity is modulated by coregulator binding. Androgen exerts their effects via the AR. The AR gene is known to be up-regulated by both estradiol via estradiol receptor α and by androgens via AR [34]. The requirement for AR-mediated gene regulation in male sex development is demonstrated by individuals with androgen insensitivity syndrome [16]. Previous study demonstrated that AR knockout mice is characterized by a defective decidual response [35], while decidualization inhibited the AR protein expression in mice uterus [36]. These conflicting results prompted us to investigate the function of AR during human decidualization. In this study, we investigated the expression of AR throughout the menstrual cycle using immunohistochemistry staining and western blot assay. The results showed a significant increase in the stroma and a decrease in the glandular epithelium in the mid-secretory phase (Fig. 10A). Western blot analysis further supported a significant decrease of AR in the endometrium during the mid-secretory phase (Fig. 10B). These results prompted us to further investigate the role of AR in the decidualization of endometrial stromal cells. The expression of AR was upregulated in PCOS endometrium (Fig. 10C, D). Bavdegalutamide (ARV-110) is an oral bioavailable specific AR degrading agent, which can lead to ubiquitination and degradation of AR. ARV-110 can effectively degrade AR. Under in vitro decidualization, PRL and IGFBP1 mRNA levels were significantly inhibited by testosterone excess, while the inhibition of IGFBP1 (53.49 ± 14.57 vs. 233.37 ± 46.64, *p* = 0.0286) and PRL (13.49 ± 4.72 vs. 239.69 ± 90.58, *p* = 0.05) was alleviated by ARV-110 treatment (Fig. 10E, F). The PDK4 mRNA and protein levels were also restored after ARV-110 treatment (Fig. 10G, H). These results suggested that the function of androgen during decidualization was elaborated through AR. Furthermore, we examined the colocalization and expression patterns of AR, SIRT1, and PDK4 in the endometrium through multiple fluorescence immunohistochemistry assays. These experiments revealed a reciprocal relationship between the expression of SIRT1/PDK4 and AR (Fig. 10I). In conclusion, our findings suggest that the SIRT1/PDK4 signaling was induced by estradiol and progesterone during decidualization, along with high glycolysis-related activity. However, the hyperandrogenic environment of endometrium inhibited the AMPK/SIRT1/PDK4 signaling via AR, decreased the levels of lactate, and resulted in an impaired decidualization in PCOS patients. A schematic pathway was proposed to illustrate the regulatory mechanism by which androgen excess regulates human decidualization via the AR/AMPK/SIRT1/PDK4 signaling pathway (Fig. 11).

## Discussion

PCOS is a mysterious and complicated endocrine disease with a combination of metabolic, reproductive, and psychological dysfunctions. PCOS-related infertility is primarily attributed to ovulation disorders/anovulation and impaired endometrial receptivity [37]. However, the alterations in the endometrium have never received commensurate attention compared to ovulatory dysfunction in PCOS. Our study suggests that women with PCOS have impaired decidualization, which may be the dominant variable in the determination of endometrial receptivity. Steroid hormones are critical for the regulation of endometrial function during the menstrual cycle [38]. Among the steroid hormone family, androgens have a beneficial effect on the regulation of decidualization and embryo implantation [39]. The purpose of this study was to further investigate the direct effects of excess androgens on the endometrium, and our results revealed that endometrial androgens were significantly higher in PCOS women, and excess androgens significantly suppress human decidualization.

Decidualization is a critical and essential process for reproduction involving a series of complex molecular and cellular events to prepare the endometrium for embryo implantation [40]. Increased lines of evidence demonstrate the significant role of impaired decidualization in the pathophysiology of PCOS [41]. This study demonstrated that the androgen excess-driven negative regulation of decidualization in the endometrium of PCOS patients is AR-dependent. Clinical evidence indicates that hyperandrogenism might contribute to PCOS pathogenesis. Kinza Younas et al. observed that the elevated androgen levels resulted in a delayed and incomplete decidual transformation of endometrial cells in PCOS patients [42]. To develop effective treatments for endometrial dysfunction in PCOS, it is important to understand how androgen-AR signaling leads to different molecular changes in the endometrium and endometrium-related reproductive outcomes in these patients. To explore the potential molecular mechanisms, biopsies at the mid-secretory phase were obtained to investigate the differentially expressed genes at the transcriptional level in PCOS patients with or without hyperandrogenism. According to the GO and KEGG analysis, a variety of enzyme proteins associated with glucose homeostasis showed abnormal changes. In particular, the hierarchical cluster analysis revealed that PDK4, which is involved in the glycolysis process, exhibited a significantly lower expression in PCOS women with hyperandrogenism. Many studies found that the flux of glucose is increased during human decidualization [43]. It has been well known that fulfilling the metabolic requirement of the embryo is important in early pregnancy [44, 45]. Previous studies have proved that glycolysis was significantly increased during decidualization [46, 47], and impaired decidualization may, in part, be attributed to the improper glucose metabolism of endometrial stromal cells [48]. Miguel A et al. demonstrated that androgen excess disturbed the development of metabolic process associated with PCOS [49]. In this study, we showed that the inhibition of androgen-induced PDK4 expression by exposure to a high concentration of testosterone could significantly suppress IGFBP1 and PRL secretion in human endometrium stromal cells, suggesting that PDK4 may play an important role in decidualization. Previous study had investigated the effects of testosterone on decidualization in the presence of 8-Br-cAMP and MPA [50], however, our study examined the effects of excess androgen on decidualization by treating decidual stromal cells for additional two days with the absence of MPA and 8-Br-cAMP. This approach may overlook the potential interactions between androgens and other decidualization-induced factors, such as 8-Br-cAMP and MPA. Several studies have demonstrated that AR could be stimulated by elevated 8-Br-cAMP levels [51, 52], which may suggest that excess androgen is not a sole factor inducing an increase in AR levels in the endometrium during decidualization. The effects of cAMP and MPA on AR levels still need further research to clarify, which is also a limitation of the treatment scheme in this study.

Pyruvate dehydrogenase kinases (PDKs) are key enzymes in mitochondria that negatively regulate the activity of the pyruvate dehydrogenase (PDH) complex through the phosphorylation of its subunit [53, 54]. Alternations in PDK expression and activity are implicated in a variety of physiological and pathophysiological processes, including insulin resistance and diabetes mellitus, cancer, and obesity [55]. Four distinct PDK isoenzymes are known to be expressed in a tissue-specific manner in mammals, with PDK4 being the predominant isoform in tissues with high energy demands, such as heart, lactating mammary gland, liver, and vascular tissue [56]. Recent studies have revealed an induction of PDK4 during human decidualization [57]. Our study also showed that the expression of PDK4, rather than other PDK family proteins (PDK1/2/3), was periodically increased during the mid-secretory phase. PDK4 was expressed in both gland and stroma in endometrium tissues, aligning with other studies examining the role of PDK in the endometrium [58, 59]. We further found that PDK4 expression is gradually increased in response to 8-Br-cAMP and MPA treatment concurrent with the onset of decidualization in a time-dependent manner. Previous studies demonstrated that aerobic glycolysis-related genes and factors are substantially induced in mice during decidualization [47]. In this study, an induction of PDK4 was also verified during mice decidualization using a pseudopregnancy mouse model. To better understand the functional role of PDK4, PDK4-siRNA and DCA were employed. Both PDK4-siRNA and DCA treatments led to a significant reduction in decidual PRL and IGFBP1 expression, perturbed cytoskeletal structure of decidualizing HESCs, and impaired uterus and deciduoma in mice, suggesting that the inhibition of PDK4 expression is a crucial step contributing to the pathogenesis of decidualization. It is worth mentioning that although we have confirmed the key role of PDK4 in decidualization, we treated HESCs with 8-Br-cAMP and MPA for 3 days, during which the expression of PDK4 did not reach its peak, which may result in less significant outcomes due to insufficient decidualization.

The results obtained in our study unveiled a novel function for PDK4 as a regulator of glycolysis. PDKs were used to be reported as the regulator of glucose utilization. DCA has been indicated with the capability of increasing mitochondrial pyruvate uptake and promoting the oxidation of glucose in the course of glycolysis [60]. In this study, we demonstrated that the level of PDK4 expression in endometrial stromal cells was significantly decreased in the PCOS group compared with the control group during the mid-secretory phase. This aligns with our previous study highlighting aberrant gene expression associated with glucose metabolism in the endometrium of PCOS patients [20]. Additionally, Zhao et al. [61] indicated that hyperandrogenism but not obesity induced the altered intermediate metabolites in classic PCOS patients by using targeted metabolomics. The present study further demonstrated that glycolysis-related gene, especially PDK4, has a lower expression in PCOS with hyperandrogenism compared with PCOS without hyperandrogenism. Moreover, androgen excess in the local endometrium could induce decreased ATP production and lactate generation, diminished glycolysis capacity, and impaired decidualization. The DHEA-induced PCOS mouse model shares hyperandrogenism, insulin resistance, altered steroidogenesis, abnormal follicular maturation, and anovulation [62]. Zhang et al. [63] have reported alterations in uterine morphology, cell phenotype, and cell function, especially in glandular epithelial cells, resulting from peripheral insulin resistance and hyperandrogenism. The present study found that DHEA-induced PCOS-like mice have impaired decidualization, which is in consistent with the previous study [64]. While previous research has predominantly focused on elucidating the impact of hyperandrogenism on granulosa cell function, follicular development, ovarian dysfunction, and ovulation [61, 65–67], our study stands out by discovering an important correlation between androgen excess, glycolysis, and decidualization within the local endometrial milium.

Decidualized stromal cells express several decidual marker genes in the implantation period in coordination with simultaneous changes in the composition of the epithelium [68]. Previous studies have emphasized the critical importance of the spatiotemporal expression of FOXO1 in orchestrating successful implantation and decidualization of endometrium stromal cells [69]. SIRT1, a widely expressed member of the Sirtuins family of Class III histone deacetylases, is known to have diverse roles in various peripheral metabolic tissues, such as liver, muscle, and adipose tissue [70]. Through epigenetic and non-epigenetic mechanisms, SIRT1 has been implicated in many biological processes, including cellular metabolism, stress response, autophagy, inflammation, and aging [30–32]. Magdalina et al. demonstrated that uterine-specific SIRT1 deficiency impairs stromal cell decidualization in mice [33]. While prior studies have demonstrated that the dysregulation of Sirtuins family members may be involved in the emergence of abnormal reproductive phenotypes, investigations into the role of SIRT1 in uterine biology have been relatively scarce [71]. It has been shown that the activation of SIRT1 facilitates FOXO1 deacetylation in the endometrium, thereby modulating the process of decidualization [33]. In this study, we demonstrated that SIRT1 was induced during decidualization, by showing that the inhibition of SIRT1 precipitates a notable decrease in PRL and IGFBP1 expression. Intriguingly, we have unveiled a heretofore unknown relationship between SIRT1 activity and PDK4 expression. Specifically, our results illustrate that the inhibition of SIRT1 activity results in a pronounced downregulation of PDK4. Our co-immunoprecipitation results further substantiate a direct physical interaction between SIRT1 and PDK4 during endometrial decidualization. It is worth noting that the PDK4 antibody failed to immunoprecipitate the SIRT1 protein, while on the contrary, the SIRT1 antibody effectively immunoprecipitated the PDK4 protein in HESCs. This phenomenon may be attributed to the low expression abundance of SIRT1 before decidualization induction, which weakens its interaction with PDK4 and makes it difficult to be detected by western blot analysis. To our knowledge, this is the first report of such an interaction between SIRT1 and PDK4 in endometrium during decidualization. However, further comprehensive investigations are warranted to elucidate the precise regulatory impact of SIRT1 on PDK4 deacetylation and to identify potential deacetylation sites on PDK4.

AMPK acts as a regulator of PDK4 in muscle and cardiomyocytes [72]. In our study, the PRL and IGFBP1 mRNA levels were significantly decreased in decidual cells after being treated by Dorsomorphin, an AMPK inhibitor. However, our results showed that A76, an AMPK activator, slightly decreased the mRNA expression of IGFBP1, which were inconsistent with previous study [57], even though IGFBP1 protein levels were increased by A76 compared with the decidualized cells. A76 is equally potent in activating the baculovirus expressed α1, β1, γ1 recombinant isoform of AMPK and inhibits dephosphorylation of Thr-172. Our western blot assay results showed that p-AMPK was significantly increased during decidualization, while the effect of A76 on activation of AMPK is not significant compared with decidualization induction alone. Previous studies have reported varied patterns of AMPK mRNA expression during decidualization, with significant upregulation observed on day 6 [73–75]. Additionally, it has been demonstrated that A76 can increase IGFBP1 and PRL mRNA expression in decidualized cells following prolonged treatment with MPA and cAMP [57]. In this study, decidualization was induced for 3 days pretreated with 8-Br-cAMP and MPA, followed by treatment with A76 for an additional 2 days, during which the decidualization agents were excluded. We speculate that the insignificant activation effect of A76 on AMPK and IGFBP1 may be attributed to insufficient duration of decidualization. These results highlight the crucial role of p-AMPK during decidualization. During in vitro decidualization, the expression of PDK4 could be significantly suppressed by AMPK knockdown, which is consistent with the previous study [57]. These results imply that the activation of AMPK has a positive regulation on PDK4 during human decidualization. Moreover, it has been investigated that the expression patterns of genes related to AMPK/ SIRT1 pathway align with those of glycolysis-related genes in various cell types, such as follicular granulosa cells and macrophages [76, 77]. Recent findings have proposed the existence of a more immediate and dynamic form of SIRT1 regulation that cells adopt under metabolic stress when changes in NAD levels are marginal or absent [78]. This rapid form of SIRT1 activation may involve the activation of AMPK and SIRT1-interacting proteins localized in the nucleus [79, 80]. Given the complex interplay between SIRT1 and AMPK, it has been difficult to untangle their roles in mediating the effects of testosterone. To provide some clarity on the correlation between AMPK and SIRT1, we performed a series of dose- and time-course experiments with testosterone during decidualization. Our results showed that both AMPK and SIRT1 mRNA levels are increased in a time-dependent manner during decidualization. Treatment of HESCs with a high dose of testosterone (10^−5^ M) significantly inhibited SIRT1 expression in an AMPK-dependent manner, suggesting that AMPK may act as the upstream of SIRT1 in decidualizing cells.

The action of androgens in the uterus is mainly mediated by its ligand AR that regulates the transcription of downstream target genes. The presence of AR in the endometrium has an important role in human reproductive function. Previous studies suggest that the androgen-induced AR activation might participate in the anti-proliferative process against estrogen and promote endometrial receptivity for embryo implantation [35]. Genetic models of loss of AR function have demonstrated that female mice with deficient AR function suffer from defective implantation and reduced fertility due to improper development of the uterus [81]. Global ARKO female mice (Ar^−/−^) display abnormal uterine growth, characterized by a significant reduction in uterine and endometrial surface area during diestrus and estrus, compared to their wild-type (WT) counterparts [82]. In humans, both insufficient and excessive circulating androgens have been associated with an increased risk of early pregnancy loss and late obstetric complication due to impaired placental function [83]. It has been previously documented that AR protein expression is upregulated in the endometrium of PCOS patients, regardless of the presence of hyperandrogenism. Elevated AR levels in the endometrium may be involved in implantation failure and recurrent miscarriage in PCOS [84]. In the human endometrium, the expression pattern of AR closely mirrors that of progesterone receptor and estrogen receptor. These receptors typically reach their highest levels during the proliferative phase of the menstrual cycle, followed by a decline in their expression as the secretory phase [85]. During early pregnancy, AR remains detectable in the stroma and decidua of the human uterus [86]. In this study, we demonstrated a decreased expression of AR in the endometrium during the mid-secretory phase, which is consistent with the previous findings. Furthermore, the results demonstrated a strong expression of AR in the endometrium of PCOS women during the mid-secretory phase. Restrain of AR activation could resume PRL and IGFBP1 secretion, which were initially suppressed by testosterone excess. It is confirmed that androgens induce the development of endometrial hyperplasia in PCOS patients through AR-mediated AMPK-α activation [87]. This study demonstrated that the expression of PDK4 downregulated by androgen excess were alleviated after treatment with ARV-110. The co-localization of AR/SIRT1/PDK4 signaling in endometrium determined by multiple fluorescence immunohistochemistry staining revealed a negative correlation between AR and both SIRT1 and PDK4 in PCOS patients. These results collectively suggest that PDK4 might be the dominant downstream target of AR signaling in endometrium. However, more research is warranted to elucidate the precise mechanisms underlying AR-mediated signaling during decidualization.

In summary, the present study provides further evidence that excess androgens play an important role in decidualization primarily by downregulating PDK4 via suppression of AMPK/SIRT1 pathway and participates in the regulation of decidualization. Further, we defined the expression and biological function of PDK4 in endometrial stromal cells during decidualization. Overall, these findings indicate that dysregulation of PDK4 in the endometrium is a novel contributor to infertility in PCOS. Targeting PDK4 may hold promise as a potential therapeutic strategy for addressing infertility in PCOS patients.

### Supplementary Information

Below is the link to the electronic supplementary material.

Supplementary file1 (TIF 3872 KB)

Supplementary file2 (TIF 4412 KB)

Supplementary file3 (TIF 28294 KB)

Supplementary file4 (DOCX 17 KB)

Supplementary file5 (DOCX 18 KB)

## Data Availability

The data sets used and/or analyzed during the current study are available from the corresponding author on reasonable request. Gene expression data were submitted to the National Genomics Data Center (NGDC) Database (Accession ID: HRA005406).

## Abbreviations

8-Br-cAMP: 8-Bromo cyclin adenosine monophosphate
AKT: Phosphorylated protein kinase B
AMH: Anti-Müllerian hormone
AMPK: AMP-activated protein kinase
AR: Androgen receptor
ARV-110: Bavdegalutamide
BMI: Body mass index
BSA: Bovine serum albumin
D1: Day 1 of pregnancy
DAPI: 4, 6-Diamidino-2-phenylindole dihydrochloride
DCA: Dichloroacetate
DEGs: Differentially expressed genes
DHEA: Dehydroepiandrosterone
DHT: Dihydrotestosterone
Dor: Dorsomorphin
Dtprp: Decidual/trophoblast prolactin-related protein
ECAR: Extracellular acidification rate
FBS: Fetal bovine serum
FOXO1: Forkhead box O1
FSH: Follicle stimulating hormone
G6PD: Glucose-6-phosphate dehydrogenase
GEO: Gene Expression Omnibus
GLUT1: Glucose transporter 1
HESCs: Human endometrial stromal cells
IGFBP1: Insulin like growth factor binding protein 1
IHC: Immunohistochemistry
IVF/ICSI: In vitro fertilization/intracytoplasmic sperm injection
LDHA: Lactate dehydrogenase A
LH: Luteinizing hormone
MPA: Medroxyprogesterone
PBS: Phosphate-buffered saline
PCA: Principal component analysis
PCOM: Polycystic ovarian morphology
PCOS: Polycystic ovary syndrome
PD1: Day 1 of pseudopregnancy
PDC: Pyruvate dehydrogenase complex
PDHE1A: Pyruvate dehydrogenase E1 alpha
PDK4: Pyruvate dehydrogenase kinase 4
PKM2: Pyruvate kinase M2
PRL: Prolactin
Prl8a2: Prolactin family 8, subfamily a, member 2
SIRT1: Silent information regulator factor 2-related enzyme 1
T: Testosterone
TCA: Tricarboxylic acid
TSH: Thyroid stimulating hormone
