# Supplementary figures and images for: Decreased AMPK/SIRT1/PDK4 induced by androgen excess inhibits human endometrial stromal cell decidualization in PCOS
Source: Cell Mol Life Sci. 2024 Jul 30;81(1):324. doi: 10.1007/s00018-024-05362-5 (PMC11335245; doi:10.1007/s00018-024-05362-5)

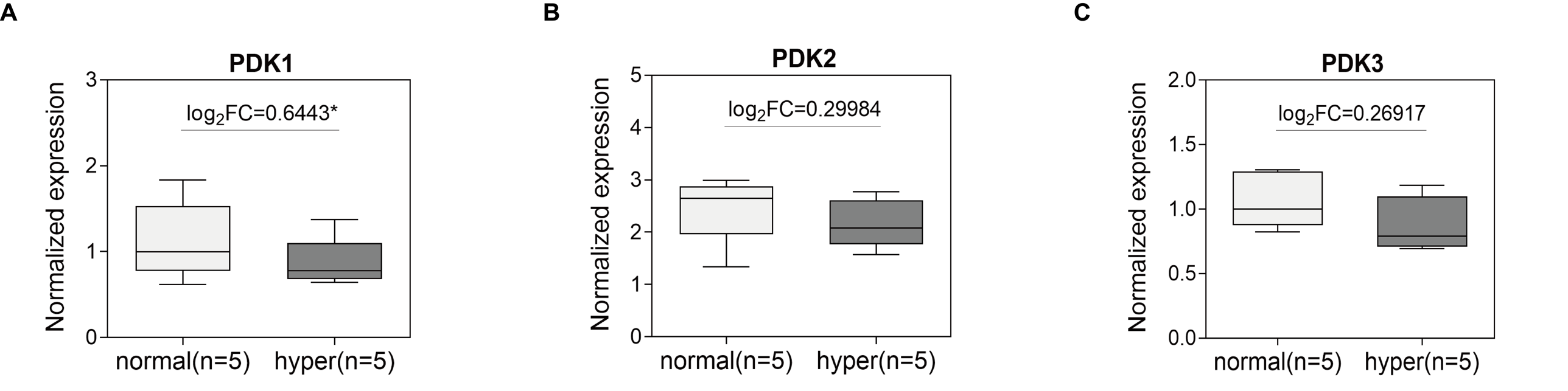

Supplement: Supplementary file 1 — Supplementary file1 (TIF 3872 KB) [file 18_2024_5362_MOESM1_ESM.tif]

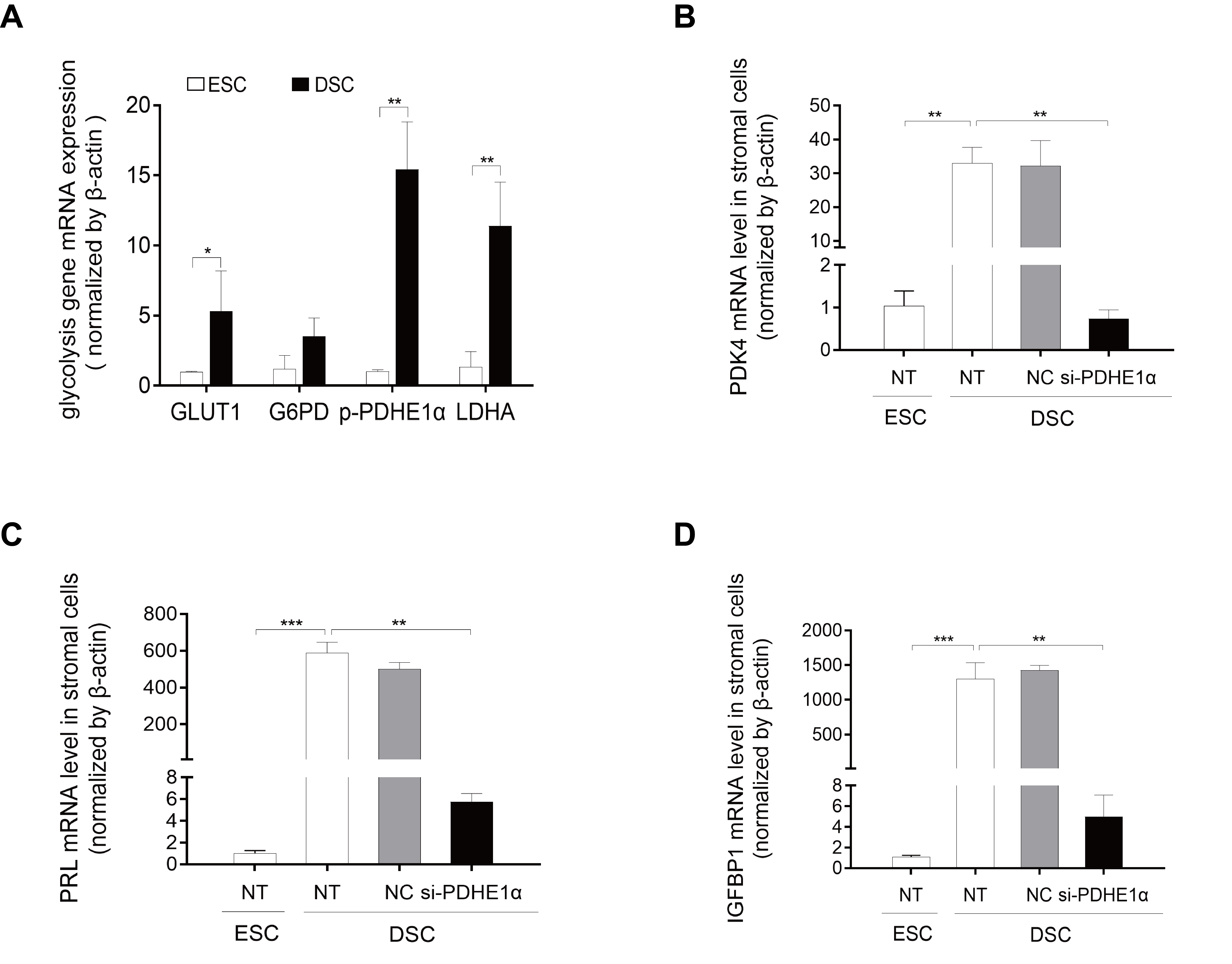

Supplement: Supplementary file 2 — Supplementary file2 (TIF 4412 KB) [file 18_2024_5362_MOESM2_ESM.tif]

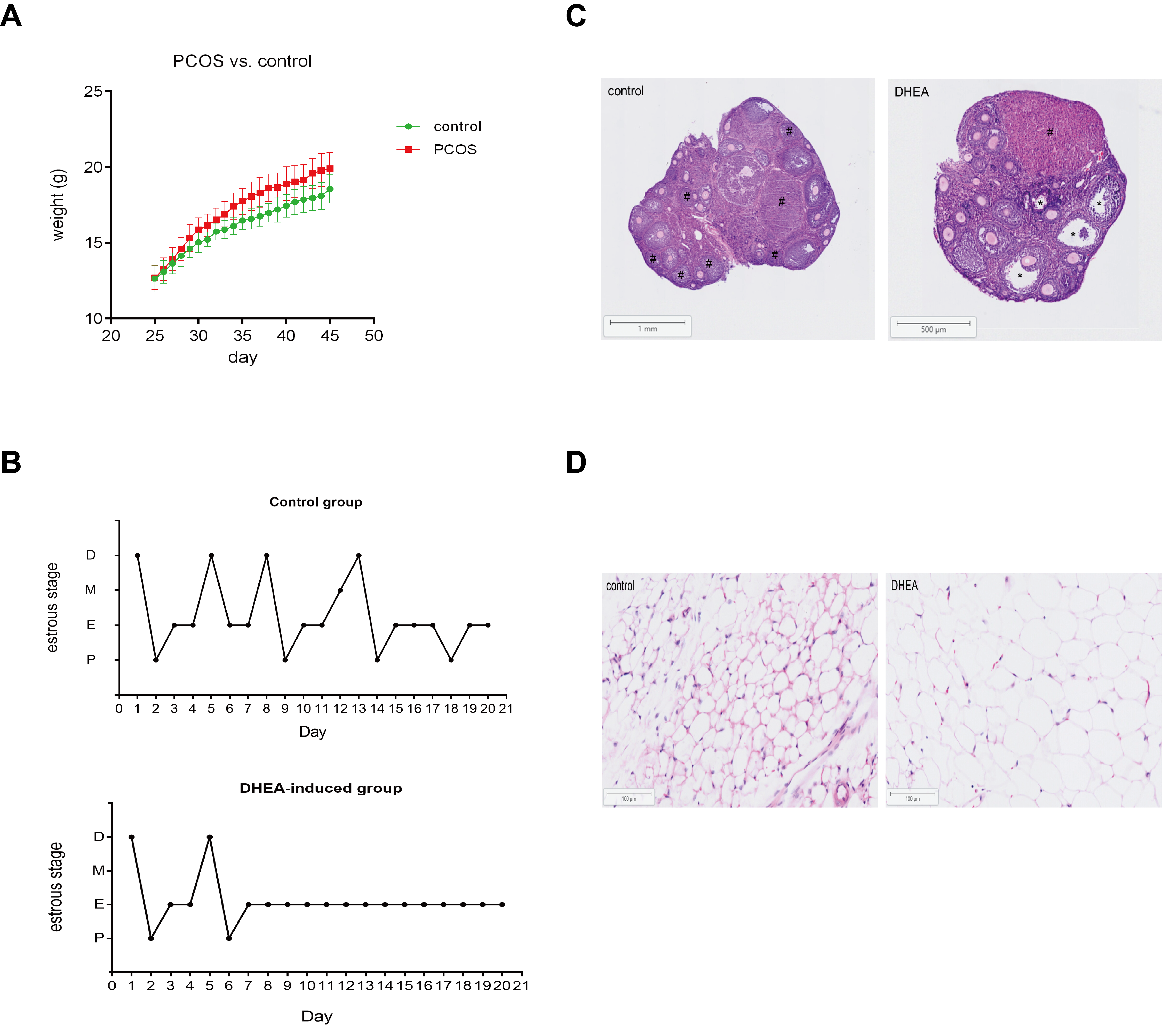

Supplement: Supplementary file 3 — Supplementary file3 (TIF 28294 KB) [file 18_2024_5362_MOESM3_ESM.tif]
