# Supplementary material for: Decreased AMPK/SIRT1/PDK4 induced by androgen excess inhibits human endometrial stromal cell decidualization in PCOS
Source: Cell Mol Life Sci. 2024 Jul 30;81(1):324. doi: 10.1007/s00018-024-05362-5 (PMC11335245; doi:10.1007/s00018-024-05362-5)
Supplement: Supplementary file 4 — Supplementary file4 (DOCX 17 KB) [file 18_2024_5362_MOESM4_ESM.docx]

| **characteristics** | **Control(n=4)** | **PCOS(n=8)** | ***p*-value** |
| --- | --- | --- | --- |
| **age (y)** | 32.0(30.0-34.0) | 30.13(27.0-33.0) | 0.236 |
| **BMI (kg/m2)** | 19.9(18.0-23.4) | 21.4(18.0-25.6) | 0.386 |
| **FSH (IU/L)** | 5.07(1.02-8.52) | 5.55(3.15-8.55) | 0.800 |
| **LH (IU/L)** | 2.53(0.39-4.79) | 8.23(2.73-14.42) | 0.004 |
| **LH/FSH** | 0.50(0.29-0.70) | 1.52(0.81-2.98) | 0.003 |
| **total T (ng/mL)** | 0.20(0.10-0.26) | 0.40(0.16-0.82) | 0.045 |
| **TSH (μIU/mL)** | 1.69(0.55-3.94) | 1.79(0.29-3.59) | 0.916 |
| **Fasting glucose (mmol/L)** | / | 5.24(4.84-5.67) | / |
| **Fasting insulin (μU/mL)** | / | 11.56(2.9-12.2) | / |
| **AMH (ng/mL)** | 3.14(2.31-3.79) | 9.23(5.80-11.15) | 0.000 |

Supplemental Table 1 Characteristics of PCOS and controls.

*Note:* BMI: body mass index; FSH: follicle stimulating hormone; LH: luteinizing hormone; T: testosterone; TSH: thyroid stimulating hormone; AMH: anti-mullerian Hormone.
