# Supplementary material for: Decreased AMPK/SIRT1/PDK4 induced by androgen excess inhibits human endometrial stromal cell decidualization in PCOS
Source: Cell Mol Life Sci. 2024 Jul 30;81(1):324. doi: 10.1007/s00018-024-05362-5 (PMC11335245; doi:10.1007/s00018-024-05362-5)
Supplement: Supplementary file 5 — Supplementary file5 (DOCX 18 KB) [file 18_2024_5362_MOESM5_ESM.docx]

Supplemental Table 2 Related primer sequences.

| Genes | Primer sequences (5′–3′) | Reverse primer (5′–3′) |
| --- | --- | --- |
| PRL | CAAAGCTGTAGAGATTGAGGAG | GTTTCAGGATGAACCTGGC |
| IGFBP1 | TTGGGACGCCATCAGTACCTA | TTGGCTAAACTCTCTACGACTCT |
| ACTB | CCTTGCACATGCCGGAG | GCACAGAGCCTCGCCTT |
| PDK1 | CACCACGCGGACAAAGG | GCCCAGCGTGACGTGAA |
| PDK2 | CCCCGTCCCCGTTGTC | TCGCAGGCATTGCTGGAT |
| PDK3 | GGAGCAATCCCAGCAGTGAA | TGATCTTGTCCTGTTTAGCC |
| PDK4 | CCGTATTTCTACTCGGATGCTG | TGGCTTGGGTTTCCTGTC |
| SIRT1 | CCCAGGGTTCAACAAATCTATGTTG | GCTTCCTAATCTCCATTACGTTGAC |
| AMPK | TGAGAAGTTCGAGTGTTCGGA | TGGTGTTTGGATTTCTGTGG |
| GLUT1 | AGAGGTTATGTGCCTGAAGTCG | GGGTGAAGGAGGAGGATGAG |
| PDHE1A | TGGAAGTGAGGAAGGAGAT | GTCGCTGGAGTAGATGTG |
| LDHA | CACCAGCAACATTCATTCCA | AGCCCGATTCCGTTACCT |
| MCT4 | CACCCACCCTCCCATTAAAGTC | CCAAGCCGCAAGGTTACAAG |
| G6PDH | CTGTGCGAGCCGTGCG | CGGGTCTGAGAGTGGG |
| Igfbp1 | GCCCAACAGAAAGCAGGAGATG | GTAGACACACCAGCAGAGTCCA |
| Prl8a2 | ACCACAACCCATTCTCAGCTGG | TGTTCAGGTCCATGAGCTGGTG |
| Dtprp | GCTGCCATTGAGTCAACCTCACTTC | ATCAACGCGTAGGCAGTGAGAAAGG |
| 18s | ATGGCCGTTCTTAGTTGGTG | CGGACATCTAAGGGCATCAC |
| β-actin | ATGCTCCCCGGGCTGTAT | CATAGGAGTCCTTCTGACCCATTC |
